# Semen Cuscutae flavonoids activated the cAMP-PKA-CREB-BDNF pathway and exerted an antidepressant effect in mice

**DOI:** 10.3389/fphar.2024.1491900

**Published:** 2024-11-25

**Authors:** Qianfeng Shao, Yue Li, Lin Jin, Sheng Zhou, Xiaowei Fu, Tong Liu, Guangbin Luo, Shaohui Du, Che Chen

**Affiliations:** ^1^ Shenzhen Traditional Chinese Medicine Hospital, Shenzhen, China; ^2^ The Fourth Clinical Medical College of Guangzhou University of Chinese Medicine, Guangzhou, China; ^3^ Centre for Translational Medicine, Shenzhen Bao’an Chinese Medicine Hospital, Guangzhou University of Chinese Medicine, Shenzhen, China; ^4^ School of Traditional Chinese Medicine, Beijing University of Chinese Medicine, Beijing, China; ^5^ School of Pharmaceutical Sciences, Guangzhou University of Chinese Medicine, Guangzhou, China; ^6^ Department of Genetics and Genome Sciences, Case Western Reserve University School of Medicine, Cleveland, OH, United States; ^7^ School of Traditional Chinese Medicine, Ningxia Medical University, Yinchuan, China

**Keywords:** depression, CUMS, cAMP, PKA, CREB, BDNF, hippocampus, metabolites

## Abstract

**Background:**

Semen Cuscutae flavonoids (SCFs) constitute a class of metabolites of Semen Cuscutae, a botanical drug that was recently found to have an anti-depression effect. This study aimed to evaluate the anti-depression effects of SCFs in chronic unpredictable mild stress (CUMS)-induced mice and to interrogate the underlying mechanisms.

**Materials and methods:**

The CUMS mice were used for assessing the effects of SCFs treatments on depression. Mice were randomly divided into five groups. Four groups were subjected to the CUMS induction and concomitantly administered orally with either the vehicle or with a high-, medium-, and low-dose of SCFs, once per day for 4 weeks. One group was kept untreated as a control. The mice were then assessed for their statuses of a number of depression-related parameters, including body weight, food intake, sucrose preference test (SPT), open field test (OFT), tail suspension test (TST), and forced swim test (FST). In addition, a day after the completion of these tests, biopsies from the hippocampus were harvested and used to perform metabolomics by HPLC-MS/MS and to assess the levels of cAMP by ELISA and the levels of PKA, CREB, p-CREB, and BDNF by Western blot analyses.

**Results:**

SCFs resulted in significant increases in both body weight and food intake and in the amelioration of the depressive-like behaviors in CUMS mice. A high-dose SCFs treatment led to significant alterations in 72 metabolites, of which 26 were identified as potential biomarkers for the SCFs treatment. These metabolites are associated with lipid, amino acid, and nucleotide metabolism. Among 26 metabolites, cAMP was positively correlated with body weight, SPT, OFT-total distance, and OFT-central residence time, while negatively correlated with immobility time in TST and FST, linking a change in cAMP with the SCFs treatment and the significant improvement in depressive symptoms in CUMS mice. Further analyses revealed that the levels of cAMP, PKA, CREB, p-CREB, and BDNF were reduced in the hippocampus of CUMS mice but were all increased following the SCFs treatments.

**Conclusion:**

SCFs could ameliorate hippocampal metabolic disturbances and depressive behaviors and cause the activation of the cAMP-PKA-CREB-BDNF signaling pathway in the hippocampus of CUMS mice.

## 1 Introduction

Depression is one of the leading causes of mental and physical disability and a significant public health challenge worldwide (WHO, 2021). Notably, adolescent depression is rising at a faster rate compared to adult depression (Avenevoli et al., 2008). The onset of depression during adolescence markedly elevates the risk of chronic recurrence, impairs lifelong functioning, and is correlated with more severe depressive episodes in adulthood (Lewinsohn et al., 1994; Weissman et al., 1999; Gollan et al., 2005; Fergusson et al., 2007; Avenevoli et al., 2008; Clayborne et al., 2019). The recurrence rate of depression following an initial episode is alarmingly high, with estimates ranging from 75% to 90% (Solomon et al., 2000; Gotlib et al., 2020). Regrettably, current therapeutic interventions remain inadequate, with over one-third of patients exhibiting resistance to existing treatments (McIntyre et al., 2023). The efficacy of these interventions is even lower for adolescent patients (Kishi et al., 2023). Additionally, a substantial portion of the currently available antidepressant medications can cause various types of side effects, such as cardiotoxicity, sexual dysfunction, and sleep disturbances (Gaynes et al., 2009). Consequently, the development of more effective antidepressant treatments remains an urgent and unmet medical need.

Historically, cyclic adenosine monophosphate (cAMP) and neurotrophic factors have been recognized as potential targets for antidepressant therapies (Duman et al., 1997). cAMP is a secondary messenger produced following the activation of certain G protein-coupled receptors (GPCRs) in response to the stimulation by specific cues, including certain neurotrophic factors. In certain brain cells, including the glial cells and neurons, the increased level of cAMP can then cause the activation of the cAMP-PKA-CREB-BDNF pathway and the accumulation of BDNF (Lessmann and Brigadski, 2009). BDNF is then secreted into the extracellular space, promoting the activation of its receptor, TrkB, through the BDNF-TrkB pathway (Tejeda and Díaz-Guerra, 2017). Remarkably, the BDNF-TrkB pathway has been identified as a key mediator of effective antidepressant therapies (Björkholm and Monteggia, 2016; Gao et al., 2022). To date, the cAMP-PKA-CREB-BDNF pathway has been the most extensively investigated signaling pathway in recent depression research, and it has been shown to play a key role in emotional regulation (Chen et al., 2024). Specifically, cAMP activates Protein Kinase A (PKA), thereby promoting the phosphorylation and activation of the CREB transcription factor and, hence, the upregulation of the expression of BDNF and other target genes (Cunha et al., 2010). BDNF plays a pivotal role in the onset, progression, and treatment of depression (Colucci-D’Amato et al., 2020). Consequently, both the BDNF-TrkB and cAMP-PKA-CREB-BDNF pathways have been the main targets for the development of novel antidepressant drugs, though progress has been limited (Björkholm and Monteggia, 2016; Gao et al., 2022). Meanwhile, it has been recognized that certain traditional Chinese medicines (TCMs) have been reported to possess antidepressant activities, while such TCM treatments presumably have favorable safety profiles (Lv et al., 2024). This realization has prompted the consideration of enhancing the efficacy of existing TCM-based antidepressant therapies as a novel strategy to develop more effective treatments with reduced risk and accelerated timelines.

*Semen Cuscuta*, the seed of the *Cuscuta chinensis* Lam plant, has been widely utilized in TCM to alleviate issues associated with female reproductive damage and male infertility for decades (Zhang et al., 2019; Liu et al., 2023). Recent research has further indicated that *Semen Cuscuta* extracts exerted an antidepressant effect on CUS mice by ameliorating synaptic structural defects and altering the composition of gut microbiota (Hou et al., 2023). Consequently, *Semen Cuscuta* has emerged as a potential candidate for developing novel antidepressant therapies.

Flavonoids are naturally occurring polyphenols that are widely present in foods and herbal supplements, including *Semen Cuscutae*. They have been demonstrated to have various pharmacological properties (Pannu et al., 2021), including antidepressant activities (German-Ponciano et al., 2018). Therefore, SCFs are hypothesized to contribute to the antidepressant effects of *Semen Cuscutae* and may thus represent a potential candidate for the development of more effective antidepressant therapies and for interrogating the mechanism(s) by which *Semen Cuscutae* exerts its antidepressant effect.

Metabolomics methodologies are increasingly employed for disease diagnosis and biomarker identification (Michely and Maurer, 2018). These methodologies aim to uncover intrinsic connections between metabolites and physiological changes, shedding light on metabolic pathways (Nicholson et al., 1999). Such studies could also be used to uncover potential biomarkers of diagnosis and/or prognosis values and to interrogate the mechanisms of the disease and the pharmacological actions of drugs (Li et al., 2010; Gao et al., 2011).

A recent study has shown that *Semen Cuscutae* extract affects the prefrontal cortex and hippocampus of depressed model mice, and its effects contribute to the antidepressant effect by altering the composition of the gut microbiota (Hou et al., 2023). However, it is known that the hippocampus is the region most closely associated with depression (Eisch and Petrik, 2012) and that depression-like behaviors are closely related to hippocampal function, with the hippocampus being a critical brain region involved in regulating emotional responses and mediating stress (Krzak et al., 2017; Lino de Oliveira et al., 2020; Idunkova et al., 2023). Thus, we then further hypothesized that the effect of SCFs on the hippocampus could also contribute to its antidepressant effect, and hence, a metabolomic analysis of the hippocampus could lead to new insights regarding the mechanism(s) by which SCFs exert its antidepressant effect. Metabolites, as the end products of cellular processes, serve as sensitive markers of physiological activity, making the hippocampus an appropriate target for metabolomic studies in depression research. Furthermore, given that CUMS mice are a well-established, widely used, and reliable model for depression research, they exhibit behavioral and physiological abnormalities similar to human depression and respond to various antidepressant treatments (Willner, 1997; Li and Liu, 2024). Thus, this study was designed to investigate the hippocampal metabolic profiles in CUMS mice, both with and without SCFs treatment, using HPLC-MS/MS and to interrogate the underlying mechanisms through which *Semen Cuscutae* exerts its antidepressant effect.

## 2 Materials and methods

### 2.1 Animal experiments

Male C57BL/6J mice (6 weeks old) weighing 20–22 g were procured from Beijing Vital River Laboratory Animal Technology Co., Ltd. (Beijing, China). They were then housed in a controlled environment with a temperature of 22°C ± 1°C, a relative humidity of 60% ± 5%, a 12-h light-dark cycle (light on between 7 a.m. and 7 p.m.) in a specific-pathogen-free animal facility, and fed a standard rodent diet.

**FIGURE 1:**
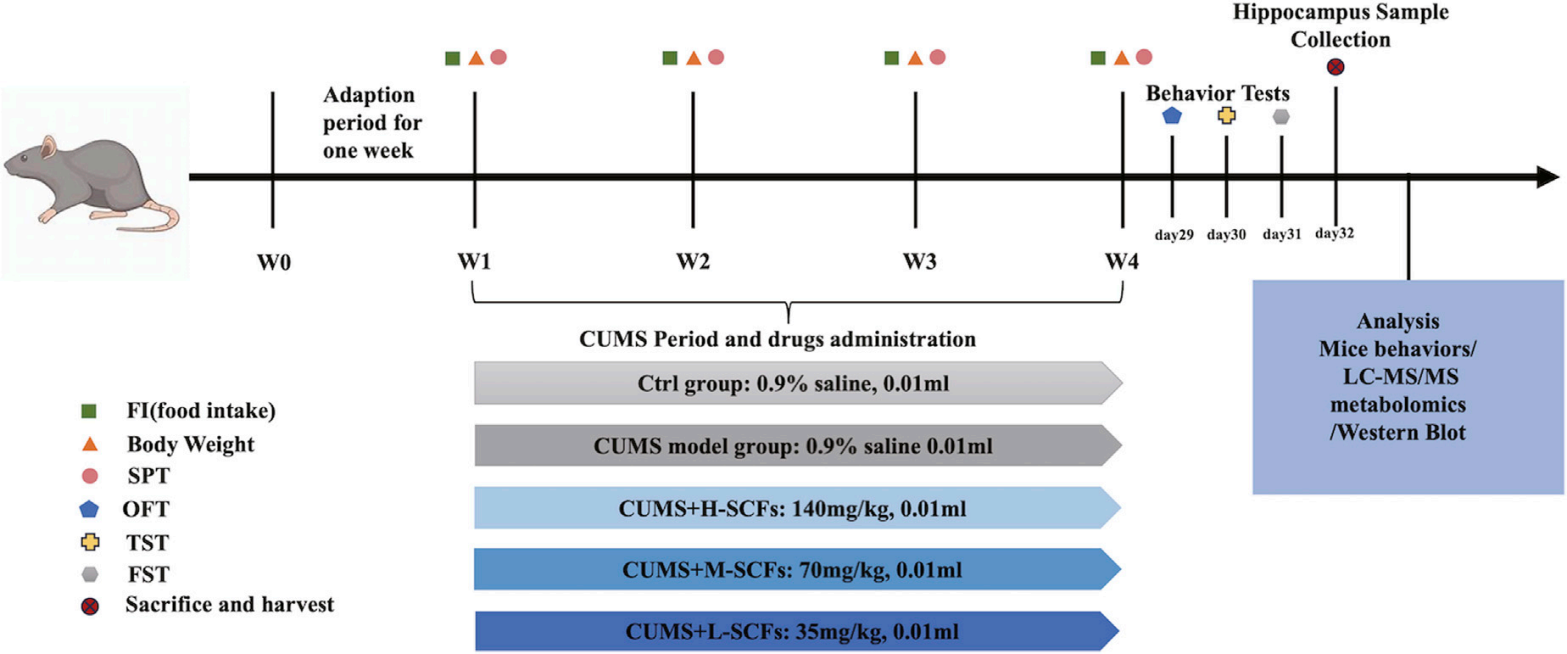
Animal experimental design.

For each series of experiments, animals were allowed to adapted for 1 week and then randomly divided into 5 groups (10 for each group). Then one group was kept as the untreated (the Ctrl group), while the other four groups were subjected to the CUMS and treated concomitantly, once per day, with either 0.9% saline as the untreated CUMS control (the CUMS group); a low dose of 30 mg/kg SCFs (the L-SCFs group), a medium dose of 60 mg/kg SCFs (the M-SCFs group), or a high dose of 120 mg/kg SCFs (the H-SCFs group), respectively. The 60 mg/kg dose is equivalent to the SCFs content estimated for the recommended human dose for *Semen Cuscutae* by the Chinese Food and Drug Administration (Xu, 2015). All animal procedures followed the National Research Council Guide for the Care and Use of Laboratory Animals. The experimental scheme is depicted in Figure 1.

### 2.2 Reagents

A single SCFs preparation was obtained from Chengdu DeSiTe Biological Technology Co. Ltd. (China). PKA and BDNF antibodies were from Pronteintech Biotech Inc., while CREB and GAPDH antibodies were from Cell Signaling Technology and Abcam. p-cAMP Response Element Binding Protein (p-CREB) was purchased from Affinity Biosciences (United States). The cAMP ELISA kit was from Rapidbio Systems, Inc.

### 2.3 Preparation of the SCFs and quantitative analysis

**FIGURE 2:**
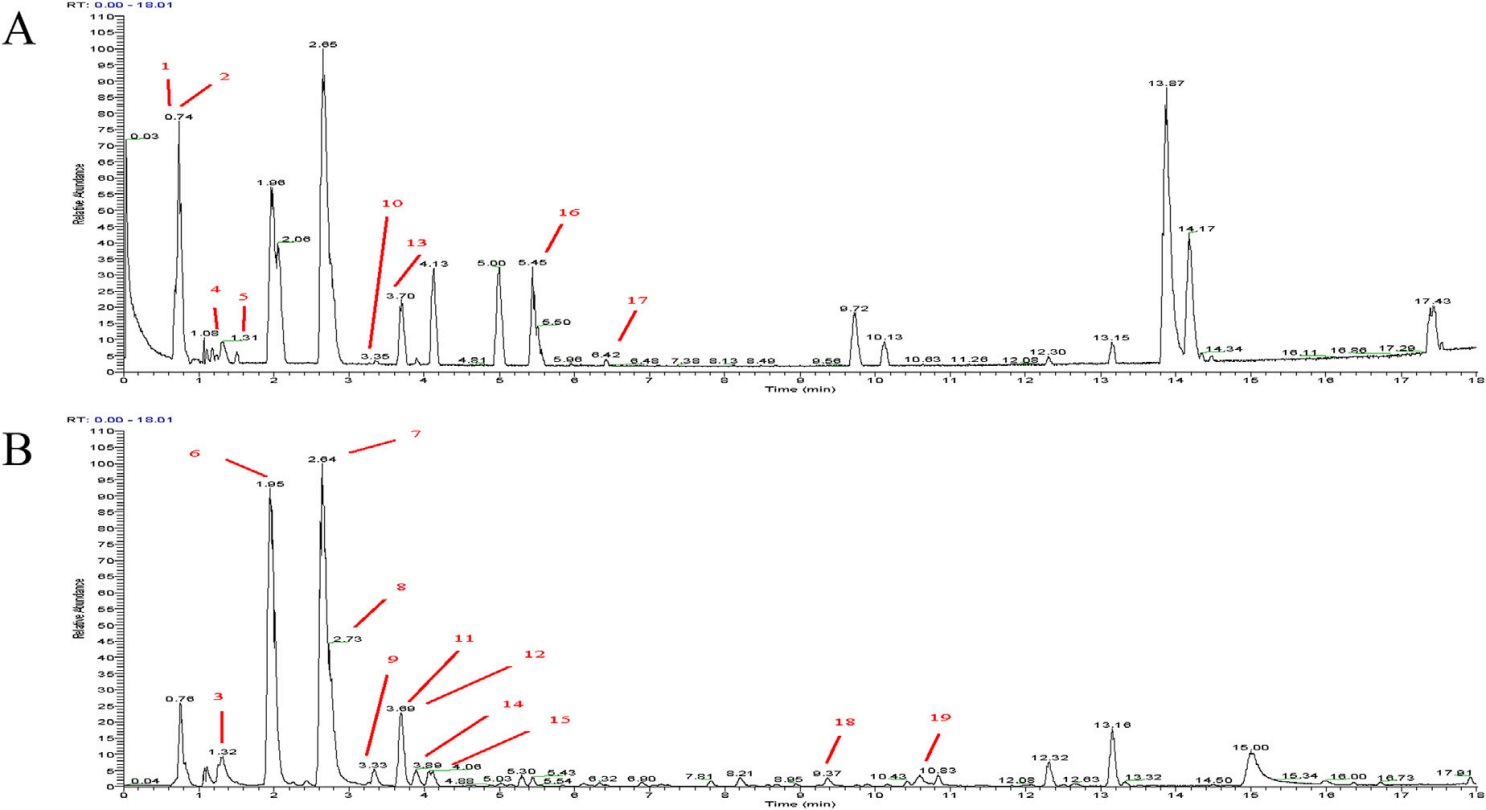
HPLC chromatogram analysis of SCFs. **(A)** Total ion current chromatogram in positive mode; **(B)** Total ion current chromatogram in negative mode. The peaks of the individual metabolites listed in Table 1 were labeled with their respective assigned numbers.

The SCFs was prepared from *Semen Cuscutae*, the dried seeds of *Cuscutae chinensis* Lam. [Convolvulaceae; Cuscutae semen] Plant in accordance with “Best practice in the chemical characterization of extracts used in pharmacological and toxicological research--The ConPhyMP--Guidelines” (Heinrich et al., 2022). Briefly, air-dried *Semen Cuscutae* was crushed and passed through a No. 4 pharmacopeia sieve. The powder was then extracted with six volumes of 90% ethanol solution at 60°C three times, 2 h each. The solutions from the three extractions were then combined and allowed to dry at 55°C until ethanol is completely removed. The crude extract was then dispersed in water and then extracted with an equal volume of dichloromethane three times. The aqueous phases were combined and passed through a AB-8 macroporous resin. The resin was then briefly rinsed with 20% ethanol and then eluted with 70% ethanol. The elution was then allowed to dry at 55°C to dryness to obtain the final product, the SCFs. The quality of the SCFs was then assessed by HPLC analyses (Waters 2,695, Waters Corporation, Massachusetts, United States). First, positive and negative mode total ion current chromatograms were established (Figures 2A, B). Then, the SCFs were analyzed using the Orbitrap Exploris 120 Quadrupole Ion Trap High-Resolution Mass Spectrometer under the following condition: Chromatographic conditions: Column: (Waters ACQUITY UPLC HSS T3 2.1 × 100 mm × 1.8 μm); Detection Wavelength: Full wavelength scan; Column Temperature: 35°C; Injection Volume: 2 μL; Flow Rate: 0.3 mL/min. The peaks of 19 metabolites including 15 flavonoids (Kaempferol 3-O-gentiobioside, Daidzin, Quercetin 3-apiosyl-(1→2)-galactoside, Quercetin-3-O-β-D-xylopyranosyl (1→6)- β-D-glucopyranoside, Hyperoside, Leucoside, Rutin, Astragalin, Quercetin-3β-D-glucoside, Quercitrin, Kaempferol, Isorhamnetin-3-O-glucoside, 3′-Methoxypuerarin, Isoorientin 2″-p-hydroxybenzoate, Tiliroside) were spotted and marked accordingly (Figures 2A, B; Table 1).

**TABLE 1:** Compound content in SCFs.

No.	Name	Formula	Mass Deviation [ppm]	Theoretical Value	tR (min)	m/z
1	Kaempferol 3-O-gentiobioside	C_27_H_30_O_16_	0.37	610.154	0.658	611.161
2	N6,N6,N6-Trimethyl-L-lysine	C_9_H_20_N_2_O_2_	−0.05	188.152	0.727	189.160
3	Chlorogenic acid	C_16_H_18_O_9_	0.75	353.087	1.320	353.087
4	Daidzin	C_21_H_20_O_9_	0.23	416.111	1.437	417.118
5	Quercetin 3-apiosyl-(1→2)-galactoside	C_26_H_28_O1_6_	−0.7	596.137	1.613	597.145
6	Quercetin-3-O-β-D-xylopyranosyl (1→6)-β-D-glucopyranoside	C_26_H_28_O_16_	−1.58	596.137	1.949	595.130
7	Hyperoside	C_21_H_20_O_12_	0.49	464.095	2.640	463.088
8	Leucoside	C_26_H_28_O_15_	−0.29	580.143	2.744	579.135
9	Rutin	C_27_H_30_O_16_	−0.68	610.153	3.337	609.146
10	Astragalin	C_21_H_20_O_11_	0.77	448.100	3.350	449.108
11	Quercetin-3β-D-glucoside	C_21_H_20_O_12_	−0.73	464.095	3.677	463.088
12	Quercitrin	C_21_H_20_O_11_	−0.81	448.100	3.691	447.093
13	Kaempferol	C_15_H_10_O_6_	−0.29	286.048	3.692	287.055
14	Isorhamnetin-3-O-glucoside	C_22_H_22_O_12_	−0.38	478.111	3.889	477.104
15	3′-Methoxypuerarin	C_22_H_22_O_10_	−0.77	446.121	4.085	445.114
16	Isoorientin 2″-p-hydroxybenzoate	C_28_H_24_O_13_	0.01	569.129	5.450	568.121
17	Tiliroside	C_30_H_26_O_13_	0.18	594.137	6.468	595.145
18	(10E,15Z)-9,12,13-Trihydroxy-10,15-octadecadienoic acid	C_18_H_32_O_5_	−0.6	328.225	9.369	327.218
19	(9Z)-5,8,11-Trihydroxy-9-octadecenoic acid	C_18_H_34_O_5_	−0.55	330.240	10.584	329.233

The Pharmacopoeia of the People’s Republic of China uses hyperoside as a quality control standard for *semen cuscutae* at the level of 0.1% or higher. In the current study, the content of hyperoside and five other main flavonoid standards (Song et al., 2015; Li et al., 2022) were chosen as the reference standards. The content of hyperoside in the SCFs was found to be 8.48%, significantly higher than the pharmacopeia’s minimum requirement of 100 μg/g (Supplementary Figure S1; Supplementary Table S1).

### 2.4 CUMS mice modeling

To establish the CUMS mouse model for depression, mice were housed individually in a one-animal per cage fashion and given randomly pairing stressors but avoided repeating on consecutive days to minimize the potential of anticipation effect for 4 weeks. The stressors included water or food deprivation (24 h), tail nip for 1 min (1 cm from the end of the tail), confinement in a tube for 2 h, white noise (85 dB, 5 min), cage shaking for 10 min, 45° cage tilting for 12 h, 24 h in damp bedding, 24 h constant light, 12 h food and water deprivation, 45°C heat stress for 5 min, swimming in 4°C cold water for 5 min (Table 2).

**TABLE 2:** CUMS modeling design.

Weeks/Days	1	2	3	4	5	6	7
1	FW + D	R + CT	I + W	C + T	CL + CS	I + O	FW + CL
2	W + T	FW + C	CL + D	CT + CS	R + I	O + T	CS + D
3	CL + I	O + W	FW + CT	R + D	C + W	R + CS	CT + T
4	C + CS	R + O	D + W	FW + O	CL + T	I + CT	R + C

The stressors were applied individually and continuously in an unpredictable and non-repetitive fashion. FW, water and food deprivation (24 h); R reversed light/dark cycle (24 h); CL, 24-h constant light; C, confinement in a tube for 2 h; I, ice water swimming (4°C, 1 min); CT, cage tilt (45°, 12 h); CS, cage shaking for 10 min; O, oven (45°C, 5 min); D, damp bedding (150 mL water +200 g bedding 24 h); W, white noise (85 dB, 5 min); T, tail pinch (1 min).

### 2.5 Behavioral tests

Monitoring tests, including the body weight (BW), food intake (FI), and Sucrose preference test (SPT), were carried out weekly. Behavioral tests, including SPT, Sucrose preference test, Tail suspension test (TST), and Forced Swim Test (FST), were performed after the 4-week treatment period.

#### 2.5.1 Body weight and food intake measurements

Body weight and food intake are significant indicators for assessing the depressive state in mice, as depressive mice often experience a loss of appetite and subsequent weight loss. Mouse body weight and food intake were measured and documented simultaneously each week of the study periods. On the first day of each week, the body weight of each mouse was determined. After that, each mouse was given a fixed amount of food at 8:00 a.m., and the leftovers were weighed at 8:00 a.m. the next day. The food intake was determined by the difference between the amount of the food provided and the amount left 24 h later.

#### 2.5.2 SPT

The SPT is employed to measure the reduction in interest in rewarding stimuli and anhedonia. Mice were individually housed and acclimated to drinking from two bottles containing a 1% sucrose solution on the first day. The subsequent day, one sucrose bottle was replaced with water, with the positions of the bottles swapped every 4 h to prevent habituation. Following a 24-h fast, each mouse was presented with two pre-weighed bottles of equal volume: one containing a 1% sucrose solution and the other pure water. Liquid intake was measured 2 h later. The sucrose preference rate was calculated using the formula: sucrose consumption/(sucrose consumption + water consumption) × 100%. The test was performed once per week throughout the entire CUMS period.

#### 2.5.3 OFT

The OFT was conducted on day 29 of the study in an open field apparatus (No. 1056025 Model: ZS-KC) with a testing area of 50 cm × 50 cm × 40 cm that are divided into 9 squares with a central square surrounded by 8 periphery squares. A 60 W white bulb was suspended 100 cm above the center of the apparatus. For each test, a mouse was gently placed in the center of the open field and allowed to explore freely for 5 min. Video tracking software (Tracking Master V3.1.62., Beijing Zhongshi Dichuang Technology Development Co., Ltd.) automatically recorded the movement of the mouse. Following the test, the total distance of movement and the time spent in the center square were determined based on the recording. To prevent any potential influence from previous trials, the four walls and the bottom of the open box were cleaned with 75% alcohol after each mouse was tested.

#### 2.5.4 TST

The TST was conducted on day 30 of the study as described with modifications. Briefly, each mouse was suspended 40 cm above the floor by its tail using adhesive tape on the edge of a rod in a visually and acoustically isolated chamber. The video tracking software (Tracking Master V3.1.62) was used to assess the duration of immobility, which was defined as the absence of escape-oriented behavior. Immobility was measured over the last 5 min of the 6-min test after a 1-min habituation period.

#### 2.5.5 FST

The Test was conducted on day 31 of the study as described with modifications. For each test. Each mouse was placed in a transparent water-containing cylinder of 45 cm in height and 20 cm in diameter with 25 cm deep water at 24°C ± 1°C. The mouse was allowed to adapt for 1 minute, and then its movement for the next 5 minutes was recorded by a video camera system (Tracking Master V3.1.62.), and the immobility time was determined based on the video. Immobility was defined as the mouse displaying complete stillness with passive floating.

### 2.6 Tissues collection

For each experiment, the animal was sacrificed on day 32 of the study (the day after the completion of all behavioral tests), and the hippocampal tissue samples were harvested, briefly frozen in liquid nitrogen, and then stored at −80°C.

### 2.7 Untargeted metabolomics

#### 2.7.1 Metabolites extraction

For each sample, 100 mg of frozen tissues were grounded with liquid nitrogen, and the homogenate was resuspended in prechilled 80% methanol with vortexing. The samples were incubated on ice for 5 min and then centrifuged at 15,000 g at 4°C for 20 min. The supernatant was diluted with water to a final concentration of 53% methanol, followed by centrifugation at 15,000 g at 4°C for 20 min. The supernatant was then used for the LC-MS/MS analysis.

#### 2.7.2 UHPLC-MS/MS analyses

UHPLC-MS/MS analyses were performed using a Vanquish UHPLC system (ThermoFisher, Germany) coupled with an Orbitrap Q ExactiveTM HF mass spectrometer or Orbitrap Q ExactiveTMHF-X mass spectrometer (Thermo Fisher, Germany) in Novogene Co., Ltd. (Beijing, China) as described. Samples were injected onto a Hypersil Goldcolumn (100 × 2.1 mm, 1.9 μm) using a 12-min linear gradient at a 0.2 mL/minute flow rate. The eluents for the positive and negative polarity modes were eluent A (0.1% FA in Water) and eluent B (Methanol). The solvent gradient was set as follows: 2% B, 1.5 min; 2%–85% B, 3 min; 85%–100% B, 10 min; 100%–2% B, 10.1 min; 2% B, 12 min. Q ExactiveTM HF mass spectrometer was operated in positive/negative polarity mode with a spray voltage of 3.5 kV, a capillary temperature of 320°C, a sheath gas flow rate of 35 psi, and an aux gas flow rate of 10 L/minute, S-lens RF level of 60, Aux gas heater temperature of 350°C.

#### 2.7.3 Metabolites identification and quantification

The raw UHPLC-MS/MS data were processed with Compound Discoverer 3.3 (CD3.3, ThermoFisher) for peak alignment, peak picking, and quantitation as described. Key parameters included peak area correction with the first QC, a mass tolerance of 5 ppm, signal intensity tolerance of 30%, and minimum intensity settings. Peak intensities were normalized to total spectral intensity, and molecular formulas were predicted based on additive ions, molecular ion peaks, and fragment ions. Peaks were matched with mzCloud, mzVault, and MassList databases for accurate identification and quantification. Statistical analyses were conducted using R (version 3.4.3), Python (version 2.7.6), and CentOS (release 6.6). Data not normally distributed were standardized by relative peak areas, with compounds having CVs over 30% in QC samples removed.

### 2.8 Measuring the levels of cAMP by ELISA

The hippocampal tissues homogenized into 10% homogenate. After centrifugation (12,000 r, 30 min), the supernatant was stored at −80°C. The level of cAMP of the supernatant was measured using a commercial ELISA kit (Rapidbio Systems, Inc.). After normalizing by total protein concentration, the relative abundance of cAMP in each sample was calculated based on the standard curve prepared in the same experiment as indicated ng/mg tissue for the brain sample. All samples were assayed in duplicate. Data were standardized to the total amount of protein.

### 2.9 Western blot (WB) analyses

Hippocampal tissues were homogenized with a RIPA lysis buffer containing protease and phosphatase inhibitors (Roche), and the BCA assay determined the protein concentration of individual samples. To perform the WB analysis, 40 µg of total proteins for each sample were separated in a 15% SDS-PAGE gel and then transferred onto a polyvinylidene difluoride (PVDF) membrane. The membrane was then incubated in 5% skim milk for 1 hour at room temperature, followed by overnight incubation at 4°C with solutions containing various types of primary antibodies including anti-PKA (1:3000), anti-CREB (1:2000), anti-p-CREB (1:500), anti-BDNF (1:2000), or anti-GAPDH (1:4000). This was then followed by an incubation with horseradish peroxidase-conjugated secondary antibodies for 1 h at 37°C. Subsequently, the membrane was incubated with an enhanced chemiluminescence substrate before being used for an X-ray film autoradiography analysis. The X-ray film was analyzed using Image-Pro Plu analysis software (version 6.0).

### 2.10 Untargeted metabolomic analyses

Metabolite identification and annotation were conducted using the KEGG, HMDB, and LIPIDMaps databases. Principal component analysis (PCA) and orthogonal partial least squares-discriminant analysis (OPLS-DA) were performed using the metabolomics data processing software metaX to determine the variable importance in projection (VIP) of metabolites. Univariate analysis (t-test) was employed to calculate the statistical significance (P-value) of metabolites and to assess the fold change (FC) between groups.

### 2.11 Statistical analyses

Statistical analyses were performed using SPSS 20 software as described. Experimental data were expressed as mean ± SD. Statistical analyses were conducted using SPSS version 20. Data are reported as mean ± SD. An independent t-test was used for equal variances, and the Mann-Whitney *U* test was used for unequal variances for two-group comparisons. For multiple group comparisons, one-way ANOVA with LSD *post hoc* was applied for equal variances and the Kruskal-Wallis test for unequal variances.

## 3 Result

### 3.1 SCFs slowed down the development of depression-like behaviors in CUMS-induced mice

**FIGURE 3:**
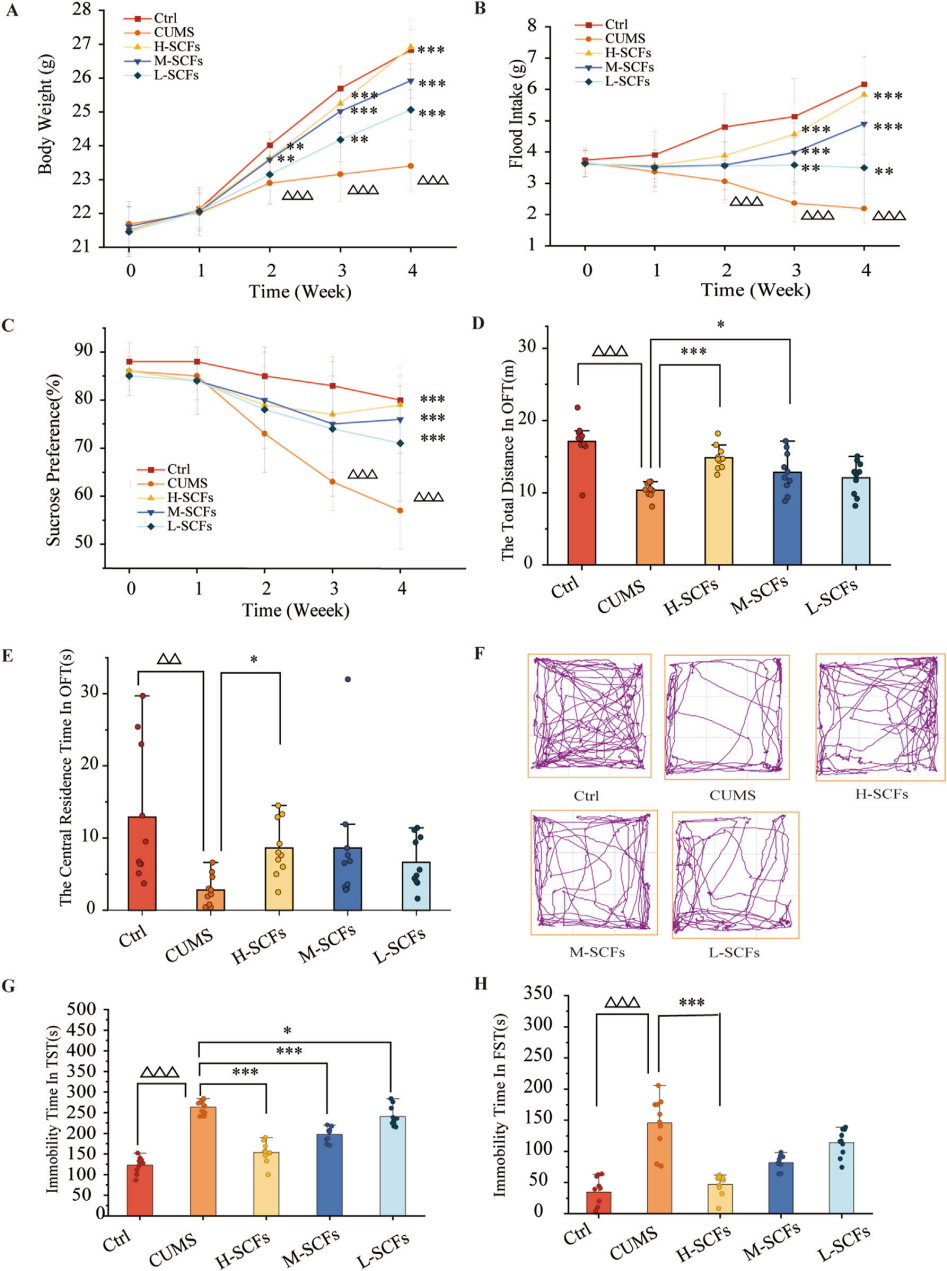
SCFs ameliorated depression-like behaviors in mice exposed to CUMS. **(A)** Weekly body weight changes during 4 weeks. **(B)** Weekly food intake changes during 4 weeks. **(C)** The relative sucrose preference rate during 4 weeks. **(D)** The total distance in OFT. **(E)** The central residence time in OFT. **(F)** The action roadmap of each group in the OFT. **(G)** The immobility time in the TST. **(H)** The immobility time in the FST. All data were expressed as means ± SEM (n = 10). ^△^ *p* < 0.05, ^△△^ *p* < 0.01, ^△△△^ *p* < 0.001 vs. the Ctrl group; *p < 0.05, **p < 0.01, ***p < 0.001 vs. the CUMS group.

Initially, preliminary experiments were performed to generate the CUMS model. The result showed that in all four experiments, the mice exhibited all the attributes expected from a CUMS model (Supplementary Tables S2–S5). These results indicated that the CUMS model could be reproducibly generated under our experimental setting. Having established the CUMS model, we then went on to perform a new set of experiments to examine the effect of SCFs treatments on the CUMS mice. Also, considering that the time of onset of each feature could be stochastic and vary among individuals or even for the same animal, it was decided that the SCFs treatments would be administered concomitantly with the stress treatments. Furthermore, the treatment time was set for 4 weeks (instead of 3 weeks in many similar studies) to approximate the chronic nature of most antidepressant treatments. The results showed no significant impacts on either body weight or food intake at Week 0 and Week 1 across the board. Starting at Week 2, however, the CUMS group exhibited a significant reduction in the degree of body weight gain compared to the Ctrl group (*p* < 0.001 at each time point). In contrast, starting at week 2, the H-SCFs and M-SCFs groups showed significantly more pronounced increases in body weight gain compared to the untreated CUMS group (*p <* 0.01, *p* < 0.001, *p <* 0.001 for CUMS + H-SCFs and *p <* 0.01, *p <* 0.001, *p* < 0.001 for M-SCFs at Week 2, 3, 4, respectively). The L-SCFs group exhibited no significance in body weight gain compared with the Ctrl group in week 2. However, it showed significantly more pronounced body weight gain in week 3 and week 4 than the Ctrl group (*p <* 0.01, *p <* 0.001, respectively). Remarkably, by week 4, the body weight of mice in the H-SCFs group was comparable to that of the Ctrl group. Thus, it appears that the SCFs treatment, particularly the high-dose SCFs treatment, was very effective in ameliorating the impairment in body weight gain in the CUMS mice (Figure 3A).

The CUMS group showed a consistent reduction in food intake at all analyzed time points. At week 1 and week 2, the SCFs treatments had a positive but not significant impact on the food intake of the CUMS mice. However, by week 3, all SCFs treatments led to significant increases in food intake in CUMS mice, and the effects appeared to be dose-dependent in nature (*p* < 0.01 for L-SCFs and *p* < 0.001 for M- and H-SCFs, respectively) (Figure 3B). Remarkably, the H-SCFs treatment resulted in nearly full normalization of all parameters tested.

Behavioral tests remain the golden standard for assessing depression-like phenotypes in animals. Thus, to determine whether SCFs indeed possesses anti-depression activities, a series of behavioral tests (SPT, OFT, TST, and FST) were also conducted alongside the physiological studies. The SPT assesses anhedonia by measuring the decreased interest in pleasurable activities like food and drink. The OFT evaluates an animal’s spontaneous locomotor activity, exploratory behavior, and anxiety in a new environment by measuring the total movement distance. Depression is associated with reduced total distance movement. The TST and FST were used to assess the state of behavioral despair and helplessness of an animal by measuring the relative immobile time, respectively.

Sucrose performance was significantly affected in the CUMS mice compared to the Ctrl group. None of the SCFs treatments significantly impacted this parameter from Week 0 to Week 2. However, beginning at Week 3, all SCFs treatments resulted in a significant reduction in the degrees of sucrose performance of the CUMS mice (*p* < 0.001 for all SCFs treatments). Thus, SCFs is effective in improving the sucrose performance defect in the CUMS mice (Figure 3C).

The results of the OFT test experiment showed that the CUMS mice exhibited significant reductions both in the total distance of movement as well as the time spent in the central area (*p* < 0.001 and *p* < 0.01, respectively). L-SCFs did not have a significant impact on these parameters. However, the M-SCFs group exhibited a significant increase in the total distance of movement only (*p* < 0.05), while the H-SCFs group showed a significant increase in both the total distance of movement (*p* < 0.001) and the time spent in the central area (*p* < 0.05) (Figures 3D–F).

Compared with the Ctrl group, the CUMS group exhibited a significant increase in the immobility time in both the TST and the FST test (*p* < 0.001, *p* < 0.001, respectively) (Figures 3G, H). All three SCFs treatments resulted in a significant reduction in the immobility time in the TST test (*p* < 0.001, *p* < 0.001, *p* < 0.05) (Figure 3G), but only the H-SCFs treatment led to a significant decrease in the immobility time in the FST test (*p* < 0.001) (Figure 3H). Thus, SCFs could improve the performance of the CUMS mice in both the TST and the FST test in a dose-dependent manner. Collectively, these results have indicated that the SCFs have a potent antidepressant activity.

### 3.2 Metabolomic analysis of the hippocampus

Having documented the effects of the H-SCFs treatment in ameliorating the various depression-like phenotypes in CUMS mice, we then went on to conduct a comprehensive LC-MS/MS metabolomic study on the hippocampus tissues of the three different groups of mice, namely, the Ctrl, the CUMS, and the H-SCFs group of mice, respectively in order to identify the metabolites that were affected by either the CUMS treatment, the H-SCFs treatment in the CUMS mice, or both.

#### 3.2.1 Quality control (QC) test

**FIGURE 4:**
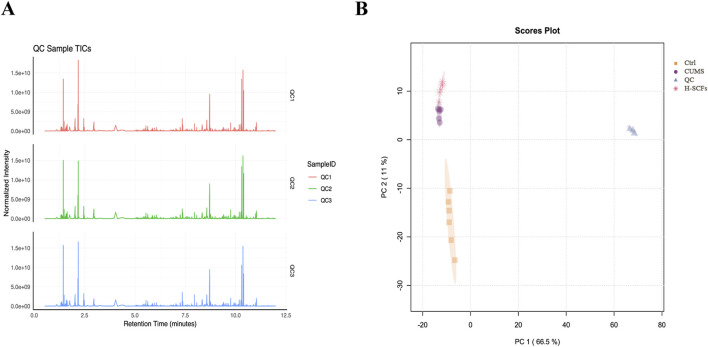
Quality control test and PCA performance test **(A)** The total ion current (TIC) maps of QC samples. The QC samples were prepared by mixing equal volumes of the experimental samples and were analyzed using the LC-MS/MS system before (Q1), during (Q2), and after (Q3) the injection of the experimental samples to assess the stability of the system throughout the entire experiment. **(B)** PCA scores for QC samples represent principal components 1 and 2.

Hippocampal metabolic profiles were analyzed using UPLC/MS in both positive and negative ion modes. Total ion current (TIC) maps of QC samples, prepared by mixing equal volumes of experimental samples, were generated before (Q1), during (Q2), and after (Q3) experimental sample injections to assess system stability and perform data quality control (Figure 4A). Principal Component Analysis (PCA) of both experimental and QC samples produced tightly clustered score plots (Figure 4B), demonstrating the system’s stability and robustness.

#### 3.2.2 PCA-score

**FIGURE 5:**
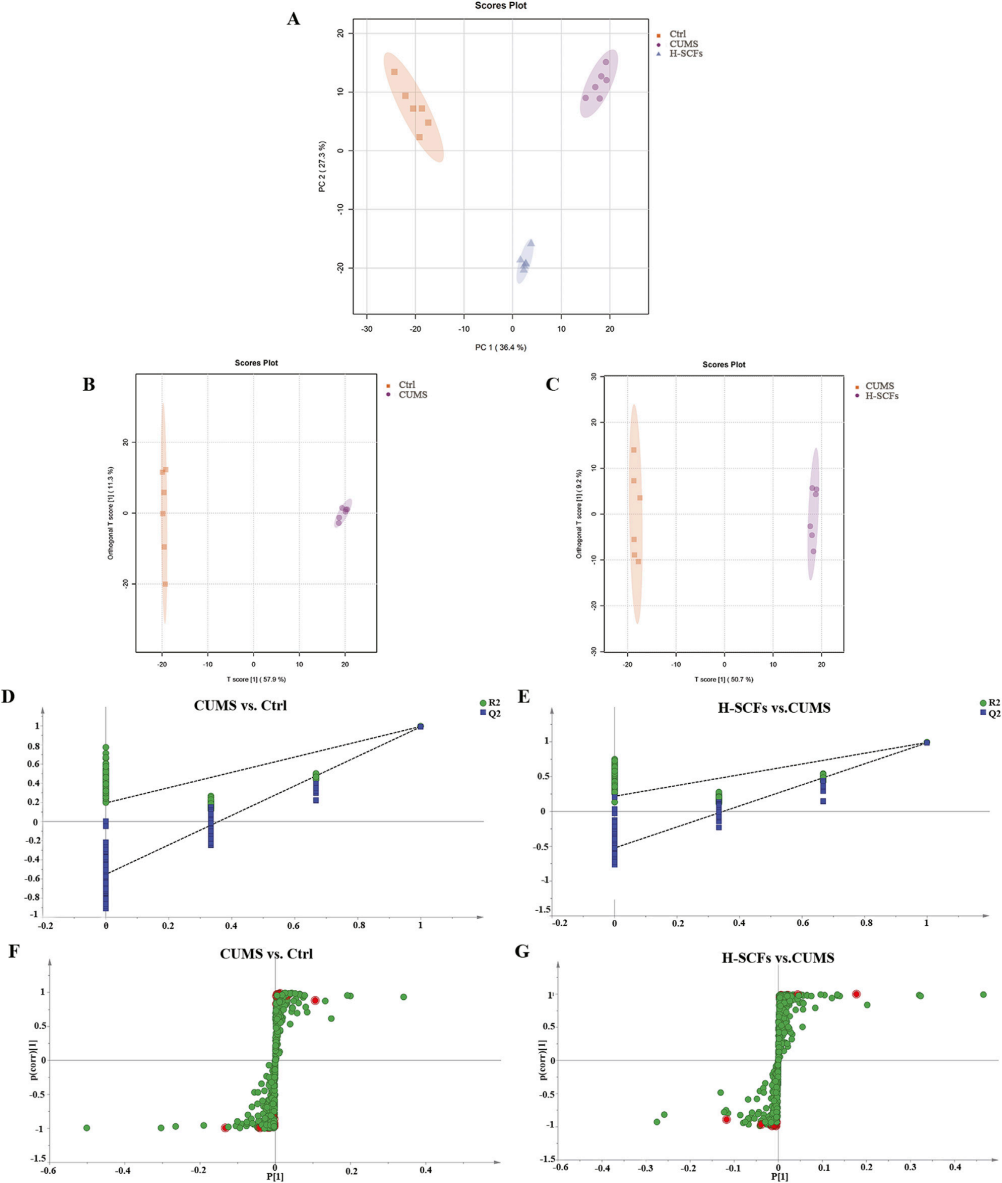
Multivariate statistical analyses of metabolites of hippocampal samples. **(A)** Hippocampal PCA score map of Ctrl, CUMS, and H-SCFs groups. **(B)** Hippocampal OPLS-DA score of CUMS and Ctrl groups. **(C)** Hippocampal OPLS-DA score of H-SCFs and CUMS groups. **(D)** OPLS-DA replacement test of CUMS and Ctrl groups. **(E)** OPLS-DA replacement test of H-SCFs and CUMS groups. **(F, G)** OPLS-DA S-plots: The 112 differential metabolites between the Ctrl and the CUMS and the 72 differential metabolites between the CUMS and the H-SCFS groups, respectively, are shown as individual dots. The 27 differential metabolites shared among the three groups were shown in red.

PCA was utilized to discern overarching distribution trends among groups and to identify and mitigate any discrete outliers. The PCA score plots demonstrated distinct separation among the Ctrl, CUMS, and H-SCFs groups, with all data clustered within the 95% confidence interval, indicating sample reliability. The H-SCFs group was located between the Ctrl group and CUMS group, with a slight bias toward the Ctrl group (Figure 5A). These data, therefore, disclosed clear-cut alterations in the hippocampal metabolite profiles among the three groups of mice.

#### 3.2.3 Identification of metabolite biomarkers in the hippocampus between groups

Orthogonal Partial Least Squares Discriminant Analysis (OPLS-DA), a supervised principal component analysis method, was used to further differentiate metabolite profiles and screen for potential biomarker metabolites between the Ctrl/CUMS pair and the CUMS/H-SCFs pair of metabolites (Figures 5B, C). The robustness and statistical significance of the OPLS-DA model were assessed by conducting 200 permutation tests (opls-perm) (Figures 5D, E). The results showed that the model had a high explanatory capacity and reliable predictive performance, with R^2^X = 0.579, R^2^Y = 0.988, and Q^2^ = 0.98 between the CUMS and Ctrl group; R^2^X = 0.507, R^2^Y = 0.994, and Q^2^ = 0.98 between the SCFs and CUMS group, respectively. The S-plot-based analyses were then carried out to identify the potential differential metabolites for each pair (Figures 5F, G). The analyses resulted in the identification of 112 and 72 metabolites for the Ctrl/CUMS pair and the CUMS/H-SCFs pair, respectively, based on a VIP >1 in the S-Plot and a t-test *p*-value <0.05 (with a fold change >2 or <0.50) (Supplementary Tables S6, S7). Among the identified metabolites, 27 were present in both data sets (Table 3), suggesting that the alterations in these metabolites were among the potential biomarkers for the pharmacological effect of the H-SCFs treatment in CUMS mice.

**TABLE 3:** Differential metabolites in hippocampal samples.

NO	t_R_ (min)	Metabolite	t_R_ (min)	Formula	Ionization mode	CUMS vs. Ctrl			H-SCFs vs. CUMS		
						VIP score	Fold change	Trend	VIP score	Fold change	Trend
1	3.02	4-oxododecanedioic acid	3.02	C_12_H_20_O_5_	POS	1.2337	10.0000	↑△△△	1.3251	0.0978	↓***
2	7.76	Tetrahydrocorticosterone	7.76	C_21_H_34_O_4_	NEG	1.1046	2.5394	↑△△△	1.3877	0.1002	↓***
3	7.27	Desoxycortone	7.27	C_21_H_30_O_3_	POS	1.2132	4.6347	↑△△△	1.3106	0.1023	↓***
4	6.91	Corticosterone	6.91	C_21_H_30_O_4_	POS	1.2538	3.4957	↑△△△	1.3581	0.1253	↓***
5	6.82	Nor-9-carboxy-δ9-THC	6.82	C_21_H_28_O_4_	POS	1.2988	5.4591	↑△△△	1.3871	0.1591	↓***
6	10.22	L-Palmitoylcarnitine	10.22	C_23_H_45_NO_4_	POS	1.1726	5.5283	↑△△△	1.2390	0.1841	↓***
7	1.58	S-Lactoyglutathione	1.58	C_13_H_21_N_3_O_8_S	NEG	1.2592	4.3534	↑△△△	1.3456	0.1884	↓***
8	9.31	Octadecanamine	9.31	C_18_H_39_N	POS	1.2489	2.9268	↑△△△	1.3497	0.1894	↓***
9	5.21	Norbuprenorphine	5.21	C_25_H_35_NO_4_	POS	1.2826	6.8324	↑△△△	1.3613	0.1980	↓***
10	9.16	CAR 20:2	9.16	C_27_H_50_NO_4_	POS	1.2490	2.4559	↑△△△	1.3534	0.2442	↓***
11	9.86	LPE O-18:3	9.86	C_23_H_44_NO_6_P	NEG	1.2836	2.4218	↑△△△	1.3726	0.4251	↓***
12	9.63	Methandrostenolone	9.63	C_20_H_28_O_2_	NEG	1.2544	2.0037	↑△△△	1.3288	0.4559	↓***
13	10.99	Stearamide	10.99	C_18_H_37_NO	POS	1.1656	2.1983	↑△△△	1.2702	0.4786	↓***
14	7.86	Chenodeoxycholic Acid	7.86	C_26_H_45_NO_6_S	NEG	1.2649	0.3456	↓△△△	1.2356	0.4921	↓***
15	5.25	Asp-Phe	5.25	C_14_H_18_N_2_O_5_	NEG	1.2092	0.4663	↓△△△	1.3093	2.0181	↑***
16	2.04	Acetyl-L-carnitine	2.04	C_9_H_17_NO_4_	POS	1.3122	0.4260	↓△△△	1.4012	2.1673	↑***
17	2.46	3-[(4-chlorobenzyl)thio]-4-methyl-5-undecyl-4H-1,2,4-triazole	2.46	C_21_H_32_C_l_ N_3_S	POS	1.1178	0.4896	↓△△△	1.3796	2.3608	↑***
18	1.51	D-Ala-D-Ala	1.51	C_6_H_12_N_2_O_3_	POS	1.2844	0.2769	↓△△△	1.2233	2.4286	↑***
19	10.48	LPC 20:2	10.48	C_28_H_54_NO_7_P	POS	1.2510	0.2868	↓△△△	1.3076	2.5600	↑***
20	1.93	Lipoic acid	1.93	C_8_H_14_O_2_S_2_	NEG	1.3019	0.3524	↓△△△	1.3895	2.6993	↑***
21	2.17	Methionine	2.17	C_5_H_11_NO_3_S	POS	1.3105	0.4444	↓△△△	1.4035	2.7344	↑***
22	5.22	FPH	5.22	C_20_H_25_N_5_O_4_	POS	1.2451	0.3767	↓△△△	1.3566	2.9465	↑***
23	5.23	GPK	5.23	C_13_H_24_N_4_O_4_	POS	1.1926	0.3909	↓△△△	1.2118	2.9718	↑***
24	5.08	T-2 Triol	5.08	C_20_ H_30_ O_7_	POS	1.0830	0.3953	↓△△△	1.3757	3.1496	↑***
25	5.07	cAMP	5.07	C_10_H_12_N_5_O_6_P	POS	1.2636	0.3212	↓△△△	1.3857	3.3252	↑***
26	1.40	Creatine phosphate	1.40	C_4_H_10_N_3_O_5_P	POS	1.2996	0.0924	↓△△△	1.2160	3.5504	↑***
27	6.46	Genistein	6.46	C_15_H_10_O_5_	NEG	1.2681	0.1966	↓△△△	1.2728	7.8461	↑***

CUMS, group vs. Ctrl group, H-SCFs, group vs. CUMS, group; ↑: upregulation, ↓: downregulation. ^△^ *p* < 0.05, ^△△^ *p* < 0.01, ^△△△^ *p* < 0.001 vs. the Ctrl group; *p < 0.05, **p < 0.01, ***p < 0.001 vs. the CUMS, group.

It was then argued that a specific change in a given metabolite is a biomarker for the effect of a given treatment of a specific disease condition if the level of the metabolite were decreased or increased in the disease condition (as compared to the corresponding normal control condition), it would be expected that its level should be changed in the opposite direction, i.e., increase or decrease, respectively. Further analysis revealed that 26 of the 27 candidates (except for Chenodeoxycholic Acid) exhibited exactly such a trend of change among the Ctrl, CUMS, and H-SCFs samples (Table 3). Specifically, for the Ctrl/CUMS pair, 14 were decreased, while 13 were increased in the CUMS data set compared to that of the Ctrl data set. Meanwhile, for CUMS/H-SCFs pair, all but one (chenodeoxycholic acid) of these 14 metabolites that were increased in the Ctrl/CUMS pair were decreased, while all 13 that were decreased in the Ctrl/CUMS pair were increased. Thus, the results further indicated that these 26 metabolites were not only a set of biomarkers for this specific depression model but also the metabolite biomarkers for the antidepressant effects of the H-SCFs treatment in this specific model.

#### 3.2.4 Bioinformatics analysis of biomarkers among three groups

**FIGURE 6:**
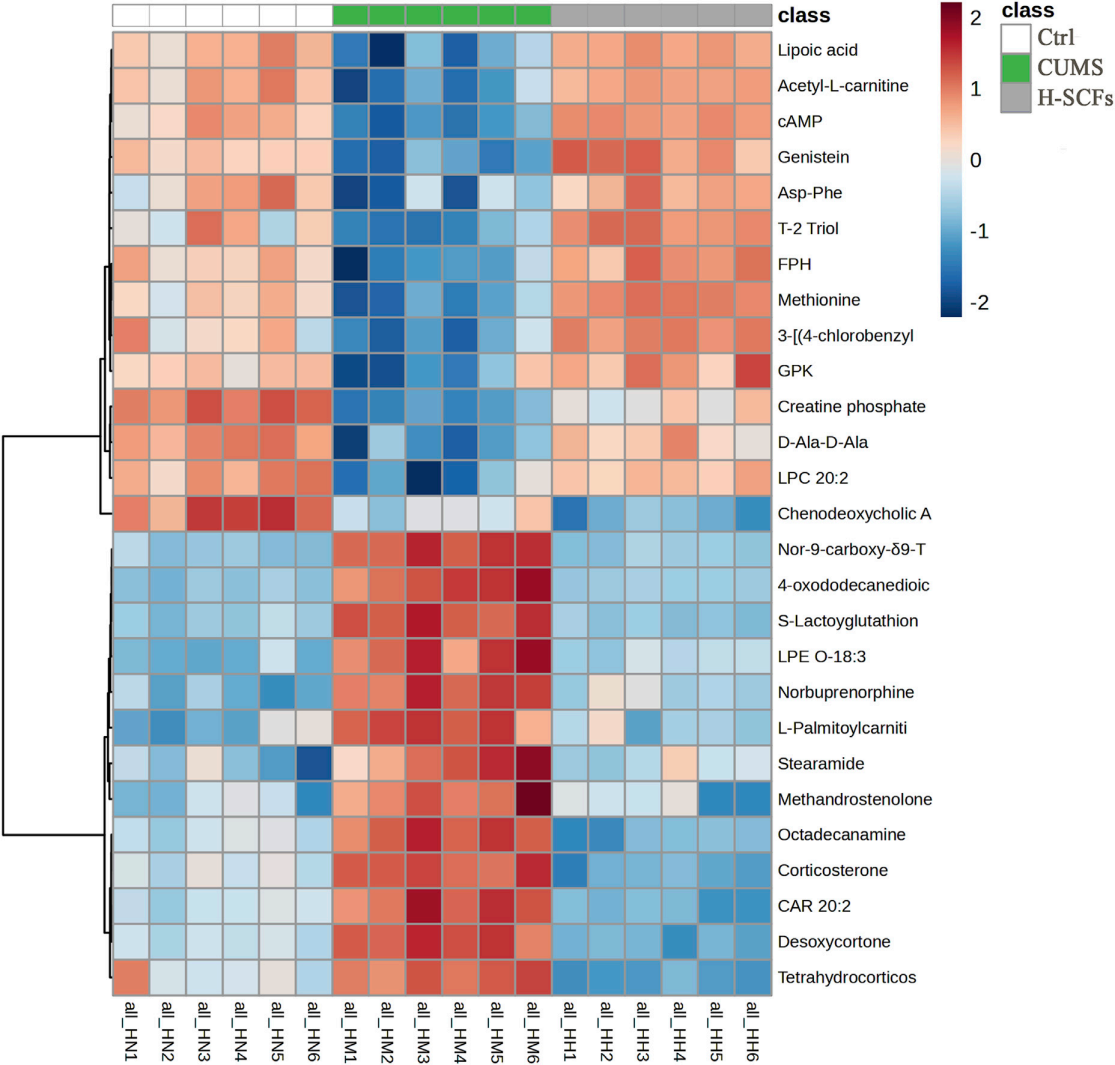
Heatmap of all potential biomarkers across all samples. This heatmap illustrates the relative levels of individual metabolites, with each column representing the metabolites from one sample and the six samples of the same group displayed together, each row representing a metabolite. The intensity of the color reflects the magnitude of the change, with values from −2 (blue, strong downregulation) to 2 (red, strong upregulation). Hierarchical clustering on the left groups’ metabolites based on their expression patterns across samples. Sample names are shown at the bottom (H1-H6, M1-M6, and N1-N6).

The newly identified biomarker metabolites were then displayed in a heat-map format based on their relative abundance and analyzed further. The results showed that the patterns within each group were relatively consistent, while significant differences were observed between different groups. Overall, there existed a significant change in the pattern both between the Ctrl and the CUMS group and between the CUMS and the H-SCFs group, except that the trends of changes were opposite between the two pairs (Figure 6). Furthermore, the 26 metabolites were found to be mainly associated with lipid and amino acid metabolism when grouped based on the types of metabolisms.

#### 3.2.5 Correlation analysis between behavior indicators and biomarkers

**FIGURE 7:**
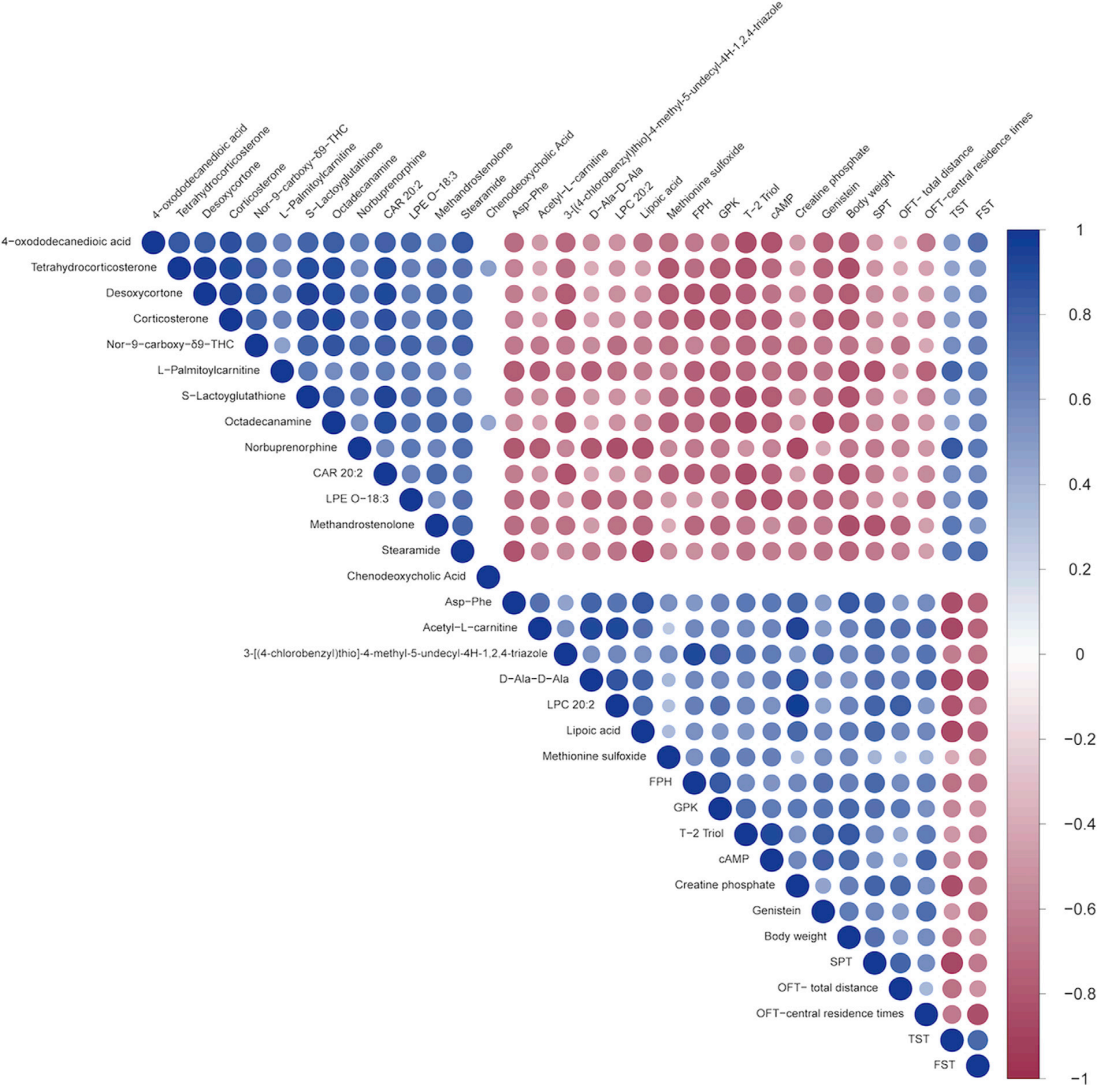
Spearman’s correlation analysis between the 27 shared metabolites and the behavioral indicators. A heatmap display of the correlation between the 27 metabolites shared by the three groups and several behavioral parameters. The positive and negative types of correlations are indicated by red and blue, respectively, while the degrees of the intensities reflect the relative degrees of correlations.

Correlation analysis revealed that 4-oxododecanedioic acid, Tetrahydrocorticosterone, Desoxycortone, Corticosterone, Nor-9-carboxy-δ9-THC, L-Palmitoylcarnitine, S-Lactoyglutathione, Octadecanamine, Norbuprenorphine, CAR 20:2, LPE O-18:3, Methandrostenolone, and Stearamide were negatively correlated with body weight, SPT, OFT-total distance, and OFT-central residence times, and positively correlated with immobility time in TST and FST. Meanwhile, Asp-Phe, Acetyl-L-carnitine, 3-[(4-chlorobenzyl)thio]-4-methyl-5-undecyl-4H-1,2,4-triazole, D-Ala-D-Ala, LPC 20:2, Lipoic acid, Methionine sulfoxide, FPH, GPK, T-2 Triol, cAMP, Creatine phosphate, and Genistein were positively correlated with body weight, SPT, OFT-total distance, and OFT-central residence times, but negatively correlated with immobility time in TST and FST. In particular, cAMP was among the metabolites that exhibited the strongest correlation with the central residence time in the OFT (cor = 0.77), followed by body weight (cor = 0.76), SPT (cor = 0.51), OFT-total distance (cor = 0.37), TST (cor = −0.55), and FST (cor = −0.66) (Figure 7). For example, the positions of five of these metabolites in the total ion current chromatograms could be readily identified (Supplementary Figure S2).

#### 3.2.6 The effects of SCFs on biological pathways in CUMS mice

KEGG pathway enrichment analysis was performed on the differential metabolites between the Ctrl and CUMS pair as well as between the CUMS and H-SCFs pair. The results revealed that the differential metabolites in both pairs were enriched in the cAMP signaling pathway. Among these, the enrichment of the cAMP pathway between the Ctrl and CUMS groups had a *p*-value of 0.06, suggesting that the cAMP pathway may play a crucial role in the pathogenesis of depression (Supplementary Table S8). Remarkably, it was noticed that cAMP was among the metabolites on this shortlist (Table 3). The levels of cAMP consistently exhibited a substantial decrease in the CUMS mice compared with those in the Ctrl mice, but the differences were diminished in the H-SCFs mice (Table 3; Figure 6). These results identified cAMP as a common metabolite that was affected both during the course of the CUMS treatment as well as in response to the H-SCFs treatment, except in the opposite direction. Then, given that cAMP is the key second messenger in the cAMP-PKA-CREB-BDNF pathway and the crucial role of BDNF in antidepressant response, it prompted the speculation that SCFs might exert its anti-depression effect by increasing the level of cAMP and hence the activation of the cAMP-CREB-PKA-BDNF pathway within the hippocampus.

### 3.3 SCFs caused the elevations of the levels of cAMP, PKA, CREB, and BDNF in the hippocampus of CUMS mice

**FIGURE 8:**
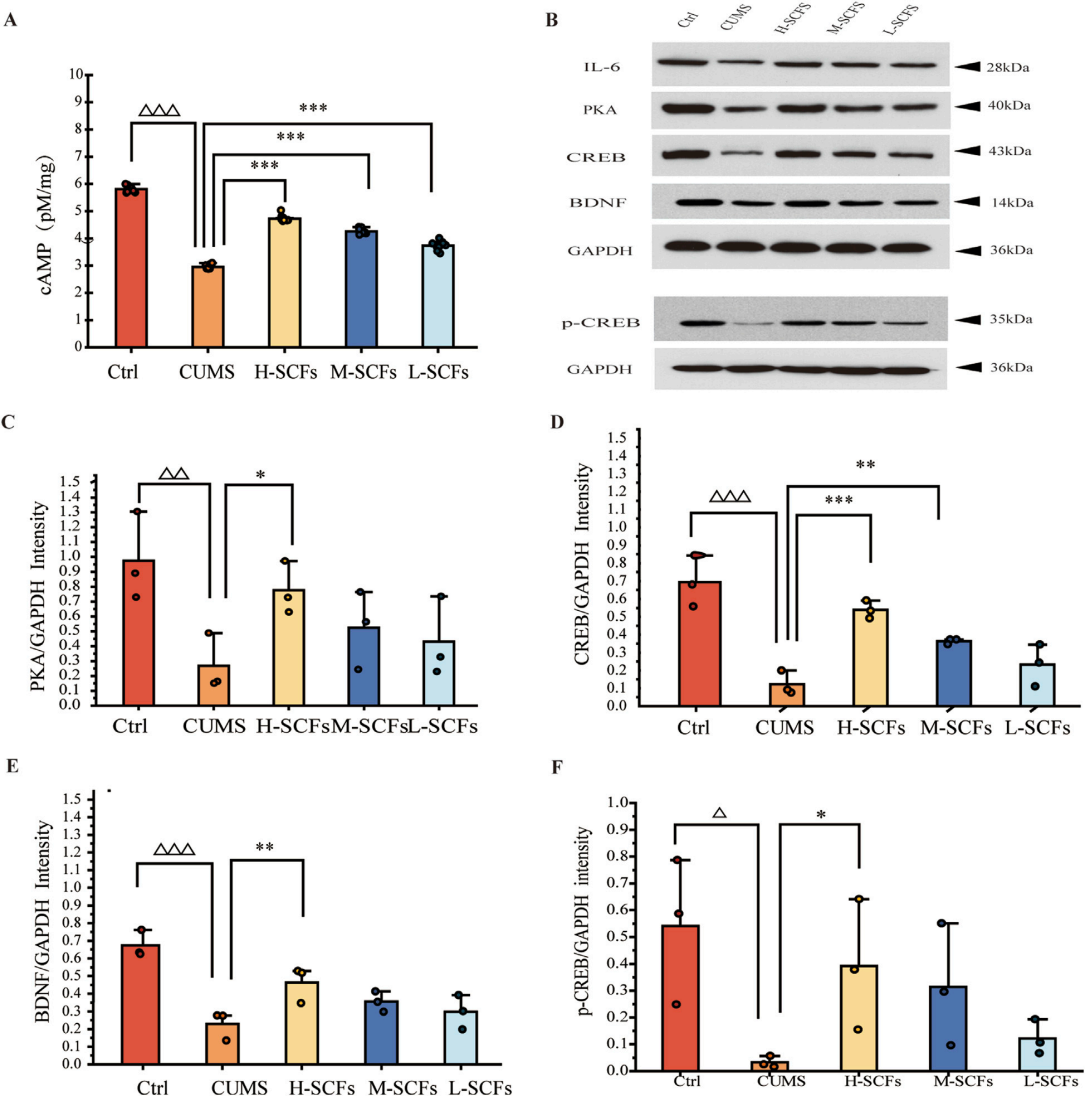
Effects of SCFs on the cAMP/PKA/CREB/BDNF signaling pathway in the hippocampus **(A)** Concentrations of cAMP in the hippocampal samples. **(B)** Representative immunoblots for PKA, CREB, BDNF, p-CREB, and GAPDH in the hippocampal samples. **(C–F)** The relative levels of PKA, CREB, BDNF, and p-CREB protein in the hippocampal samples. All data were expressed as means ± SEM (n = 3). ^△^ *p* < 0.05,^△△^ *p* < 0.01,^△△△^ *p* < 0.001 vs. the Ctrl group; *p < 0.05, **p < 0.01, ***p < 0.001 vs. the CUMS group.

To assess whether SCFs caused the elevation of cAMP by promoting the activation of the cAMP-PKA-CREB-BDNF pathway in the hippocampus, the levels of the key components of this pathway in hippocampus tissue samples were analyzed. The level of cAMP was decreased in CUMS mice compared with Ctrl mice (*p* < 0.001). Treatments of CUMS mice with SCFs significantly increased the levels of cAMP (*p* < 0.001, *p* < 0.001, *p* < 0.001 for H-, M- and L-SCFs, respectively) (Figures 8A, B). Meanwhile, the protein levels of PKA, CREB, p-CREB, and BDNF in the hippocampus were significantly decreased in CUMS mice compared with the Ctrl mice (*p* < 0.01, *p* < 0.001, *p* < 0.05, *p* < 0.001, respectively) (Figures 8B–F). The expression of PKA and BDNF was significantly upregulated by only the H-SCFs treatment (*p* < 0.05, *p* < 0.01) but not the L-SCFs and M-SCFs treatments. However, both the H-SCFs and M-SCFs significantly increased the levels of expressions of CREB (*p* < 0.001, *p* < 0.01, respectively), while the L-SCFs treatment had no significant impact on the levels of CREB expression (Figures 8C–E). On the other hand, high-dose SCFs resulted in a significant increase in the levels of the CREB that is phosphorylated at its serine-133 residue (p-CREB) (*p* < 0.05) (Figures 8B, F). Thus, these data showed that the cAMP-PKA-CREB-BDNF could be activated by the SCFs treatment, particularly the H-SCFs treatment.

## 4 Discussion

This study was based on the hypothesis that SFCs could be an excellent candidate for the rapid development of new antidepressant drug(s) and therapies and that a proper metabolomic study could aid in the identification of metabolites as candidates for antidepressant treatments based on such drug(s) and providing new insights regarding the corresponding antidepressant mechanism(s). We first assessed the antidepressant effects of three distinct doses of SCFs treatments on CUMS mice over an extended 4-week treatment period (Previously, a study with a 3-week treatment of Semen Cuscutae extract one CUS mice has been reported) (Hou et al., 2023). The three different SCFs treatments are of high, medium, and low doses of SCFs (H-SCFs, M-SCFs, and L-SCFs), respectively, with the M-SCFs dose as the mouse equivalent dose for the SCFs content in the corresponding *Semen Cuscutae* dose recommended for humans. Intriguingly, time-course studies revealed that full normalization or near full normalization effects on the three pathophysiological parameters were observed only at the 4-week point of the H-SCFs treatment, indicating that the equivalent dose is not optimal and that the extended treatment is essential for achieving the optimal beneficial effect. Furthermore, the near-full normalization of certain depressive phenotypes suggests that SCFs has a potent antidepressant activity and could represent a promising candidate for the development of more effective antidepressant therapies.

Specifically, during the 4 weeks of CUMS exposure, SCFs treatment was concurrently administered while measuring body weight, food intake, SPT, OFT, TST, and FST to evaluate depressive symptoms and the therapeutic effects of SCFs. Four weeks later, mice showed reduced body weight, food intake, anhedonia, decreased exploratory behavior, and increased behavioral despair, mirroring clinical depression symptoms (Hill et al., 2012; Willner, 2017). These findings indicated that the depression model was successfully established and consistent with previous studies (Bagot et al., 2015; Li et al., 2023; Yao et al., 2023) and with our preliminary experiment results (Supplementary Tables S2–S5). High-dose SCFs significantly improved all stress-induced changes, while medium and low doses exhibited less pronounced effects on body weight, food intake, anhedonia, and despair behavior, suggesting a dose-dependent nature of the antidepressant activity.

Additionally, mice aged 6–13 weeks serve as a model for the chronic stress experienced during adolescence (Flurkey et al., 2007). Depression-like behaviors are closely related to hippocampal function, with the hippocampus being a critical brain region involved in regulating emotional responses and mediating stress (Krzak et al., 2017; Idunkova et al., 2023). Thus, the study design should be well suited for a study aiming at learning new insights into antidepression mechanisms via a metabolomic study of the hippocampus.

The metabolomic study was meticulously designed to include six animals per treatment group and was executed with high precision throughout. Notably, all 26 potential biomarkers exhibited differential expression between Ctrl mice and CUMS mice, as well as between untreated CUMS mice and those treated with high-dose SCFs. These changes were characterized by opposite trends in the levels of each biomarker, predominantly involving lipid metabolism, amino acid metabolism, and nucleotide metabolism, as revealed by pathway enrichment analysis. Consequently, they represented a set of valid metabolite biomarkers for the effects of H-SCFs treatment in CUMS mice and potential candidates for biomarkers indicative of antidepressant efficacy in humans.

Lipid metabolism is essential for maintaining cell membrane integrity and signaling pathways, often disrupted in depressive disorders. Research has demonstrated alterations in lipid profiles within the hippocampus of depressed individuals, particularly in phospholipids and fatty acids, which can influence neurotransmitter function and neuroinflammation, thereby contributing to the pathophysiology of depression (Colin et al., 2003; Tracey et al., 2021). Patients with depression exhibit lipid metabolism abnormalities, with those experiencing suicidal tendencies showing even more pronounced disturbances (Capuzzi et al., 2020). Elevated corticosterone (CORT) levels have been shown to induce hippocampal atrophy, impair neurogenesis, and reduce synaptic plasticity, all of which contribute to the cognitive and emotional symptoms of depression. The hippocampus is particularly susceptible to the effects of elevated corticosterone, which can lead to reductions in hippocampal volume observed in depressed patients. Additionally, elevated corticosterone levels resulting from chronic stress have been shown to reduce BDNF expression in the hippocampus, thereby weakening its neuroprotective function (Smith and Cizza, 1996; Schaaf et al., 1998). In this study, SCFs were observed to increase both CORT and BDNF expression levels. Desoxycortone (DC), also known as desoxycorticosterone (DOC), is an adrenal cortex hormone with mineralocorticoid effects similar to aldosterone. DC, a corticosteroid, has been associated with alterations in mood and behavior. Its effects on depression are complex, varying with dosage and individual response (Brown et al., 2007).

Amino acid metabolism is a crucial metabolic pathway in the body, with various amino acids contributing differently to the development of depression. Research suggests that dysregulation of amino acid metabolism is frequently observed in depression, with altered levels of these amino acids potentially serving as clinical biomarkers for the condition (Lu et al., 2014; Baranyi et al., 2016). Some amino acids are also known as the precursors for neurotransmitters in the central nervous system (Rai et al., 2005). For example, methionine serves as a precursor to S-adenosylmethionine (SAMe), which is involved in the methylation of neurotransmitters, including serotonin, dopamine, and norepinephrine. These neurotransmitters play a critical role in mood regulation, and their imbalance is frequently associated with depression. Additionally, SAMe, both as a monotherapy and as an adjunct to other antidepressants, has been demonstrated to have antidepressant effects in Major Depressive Disorder (MDD) (De Berardis et al., 2016; Sharma et al., 2017).

Creatine phosphate (PCr) plays a role in energy metabolism within the brain. Given the potential mitochondrial dysfunction and disturbances in energy metabolism observed in depression, creatine phosphate may ameliorate these symptoms by enhancing intracellular ATP availability. This improvement could support normal neuronal function, including neurotransmitter synthesis and release. Indeed, it has been shown that creatine supplementation could elevate PCr levels in the brain, mitigate neurotransmitter depletion, and potentially exert neuroprotective effects through anti-inflammatory and antioxidant mechanisms (Kazak and Cohen, 2020).

Cyclic adenosine monophosphate (cAMP) can be regarded as a by-product of nucleotide metabolism. However, under normal physiological conditions, cAMP is primarily generated through the activation of adenylyl cyclase. Activation of certain G protein-coupled receptors (GPCRs) serves as a primary mechanism for stimulating this enzyme, thereby promoting cAMP production. Subsequently, cAMP can activate PKA, CREB, and BDNF in a sequential manner, completing the cAMP-PKA-CREB-BDNF signaling cascade. BDNF, a critical neurotrophic factor, is essential for neuronal survival, regeneration, synaptic plasticity, and cognitive functions, all of which may be compromised in depressive disorders (Xue et al., 2016; Chakrapani et al., 2020). Our result also showed that cAMP was positively correlated with body weight, SPT, OFT-total distance, and OFT-central residence times and negatively correlated with immobility time in TST and FST, indicating that cAMP plays a crucial role in alleviating depressive symptoms.

An increase in cAMP levels following antidepressant treatments in the hippocampus of depressive animals or humans has been well-documented over time (Duman et al., 1997). Antidepressants, such as SSRIs, elevate cAMP levels, thereby activating the PKA-CREB-BDNF pathway, which enhances BDNF expression and is associated with improved mood and cognitive function (Luo et al., 2017). Significantly, activation of this pathway has been linked with the effectiveness of antidepressant treatments (Nibuya et al., 1996; Xue et al., 2016; Fujita et al., 2017; Luo et al., 2017; Yang et al., 2019). The cAMP-PKA-CREB-BDNF pathway functions upstream of the BDNF-TrkB pathway, which mediates the antidepressant effects of various treatments (Wang et al., 2019). Therefore, it is plausible that the elevation of cAMP levels could exert antidepressant effects. Importantly, our data demonstrated that the levels of several key components of the cAMP-PKA-CREB-BDNF pathway, including cAMP, PKA, CREB, and BDNF, were reduced in the hippocampal tissues of CUMS mice compared to normal controls. Remarkably, however, these levels were elevated following treatment with H-SCFs. Intriguingly, previous research has shown that hyperoside, a known metabolite of SCFs, can activate the BDNF-TrkB pathway (Björkholm and Monteggia, 2016; Gong et al., 2017). In this context, it is noteworthy that the current study is the first to extend the antidepressant effects of SCFs, and by extension, Semen Cuscutae, to the cAMP-PKA-CREB-BDNF pathway. However, it remains unclear whether this outcome is a direct result of pathway activation or an indirect consequence mediated by the cAMP-PKA-CREB-BDNF pathway. Nevertheless, these findings collectively suggested that activation of the cAMP-PKA-CREB-BDNF pathway could be a critical mechanism through which SCFs exert their antidepressant effects, and according to safe and effective antidepressant drugs could be developed by targeting factors upstream of the cAMP-PKA-CREB-BDNF pathway with this specific metabolite or certain metabolite(s) thereof.

Collectively, the results of the metabolomic study revealed that SCFs may influence various categories of metabolites potentially relevant to the mechanisms of antidepressant treatments in the hippocampus. This finding aligns with the current understanding that multiple aspects of antidepressant effects involve several brain regions in both rodents and humans (Delaveau et al., 2011; Yang et al., 2023; Lynch et al., 2024). Remarkably, the result of the current study has shown that the H-SCFs treatment could affect these multiple categories of metabolites, suggesting its effects on more than one metabolic and biological pathway. In this regard, it remains unclear whether these effects were due to the direct effects of SCFs treatment on multiple pathways in the cells within the hippocampus or as the results of the sequential effects of a single initial response on multiple pathways. Similarly, further research is required to identify the specific metabolite(s) of SCFs that are responsible for these effects.

Such information will prove valuable for developing new antidepressant drugs in the future.

Numerous botanical drugs have been reported to exhibit antidepressant activity to date (Lv et al., 2024). SCFs could not only lead to the reversal of every depressive-like feature in the CUMS mice examined but also resulted in the activation of the cAMP-PKA-CREB-BDNF pathway. Such a comprehensive improvement in depressive features by a single extract is highly desirable in the context of drug development. Furthermore, as mentioned earlier, the antidepressant effect of the M-SCFs treatment, which represents the human-equivalent dose in mice, was inferior to that of H-SCFs at a dose twice as high. Thus, even if the currently recommended dose of Semen Cuscutae-based treatment proves effective as an antidepressant, there is likely room for improvement. Moreover, SCFs could prove superior compared to Semen Cuscutae in drug development. In this regard, it is worth pointing out that the H-SCFs treatment did not cause any significant distress in CUMS mice. Thus, it is possible that highly effective antidepressant treatments could be developed with SCFs. Once again, given the established safety record of Semen Cuscutae and the feasibility of developing TCM drugs from herb extracts such as SCFs, the associated drug development process should involve reduced risk and proceed at a more accelerated pace.

The current study has some limitations. First, the study is limited to the use of a single preclinical model of depression. Second, the study could have included experiments to determine whether the antidepressant activity of the SFCs treatments is really based on its ability to activate the cAMP-PKA-CREB-BDNF pathway, for example, by assessing whether the antidepressant effect of the H-SFCs treatment would be abolished or significantly attenuated following an interruption or blockage of the pathway either via a genetic (gene knockout) approach or by using various agents that could block the pathway.

## 5 Conclusion

SCFs could ameliorate hippocampal metabolic disturbances and depressive behaviors in CUMS mice and cause the activation of the cAMP-PKA-CREB-BDNF signaling pathway in the hippocampus.

## Data Availability

The original contributions presented in the study are publicly available. The study is now identified in the MetaboLights repository, accession number MTBLS11688. Further inquiries can be directed to the corresponding author/s.

## Abbreviations

SCFs, Semen Cuscutae flavonoids; CUMS, Chronic unpredictable mild stress; cAMP, Cyclic Adenosine monophosphate; PKA, Protein kinase A; CREB, cAMP-response element binding protein; p-CREB, phosphorylated cAMP response element-binding protein; BDNF, Brain-derived neurotrophic factor; FST, Forced swimming test; OFT, Open field test; SPT, Sucrose preference test; TST, Tail suspension test; CP, Creatine phosphate; SAMe, S-adenosylmethionine; MDD, Major Depressive Disorder; CDCA, Chenodeoxycholic Acid; DC, Desoxycortone; CORT, corticosterone; PCr, Creatine phosphate; PCA, Principal Component Analysis; OPLS-DA, Orthogonal Partial Least Squares Discriminant Analysis.
