# Supplementary material for: Semen Cuscutae flavonoids activated the cAMP-PKA-CREB-BDNF pathway and exerted an antidepressant effect in mice
Source: Front Pharmacol. 2024 Nov 25;15:1491900. doi: 10.3389/fphar.2024.1491900 (PMC11625582; doi:10.3389/fphar.2024.1491900)
Supplement: Supplementary file 1 [file DataSheet1.docx]

Supplementary Materials

**1.1 Supplementary Figures**

**Supplementary** **Figure 1.**


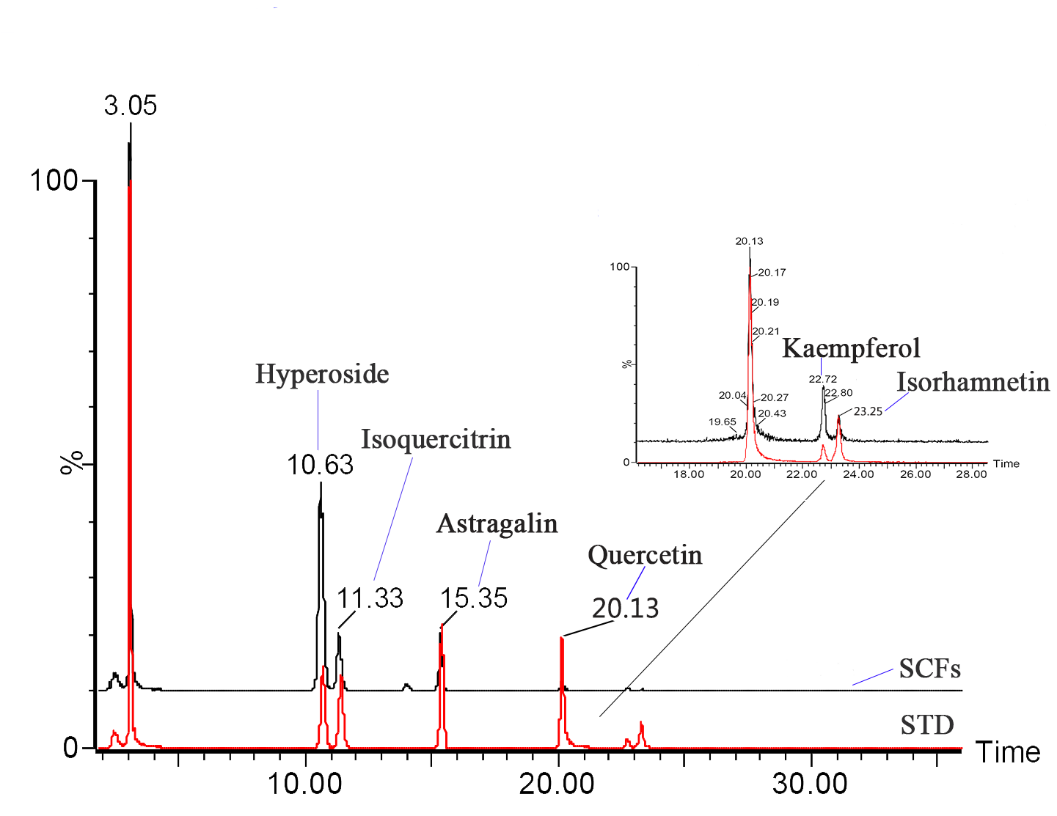


**Supplementary Figure 1: Comparative LC-MS Chromatograms of Six Flavonoid Standards and SCFs.** Red peaks represent the six flavonoid standards (Hyperoside, Isoquercitrin, Astragalin, Quercetin, Kaempferol, Isorhamnetin), while black peaks correspond to the SCFs sample. The overlay allows for the comparison of retention times and the identification of these flavonoids in the SCFs.

Six flavonoid standards were analyzed using a Waters TQ-S micro HPLC-MS system, as detailed in Supplementary Table 1. Chromatographic conditions were as follows: Column: Waters HSS T3, 2.5 μm, 3.0 × 150 mm; Detection: full wavelength scan; Column temperature: 40°C; Injection volume: 2 μL; Flow rate: 0.5 mL/min.

**Supplementary Figure 2**


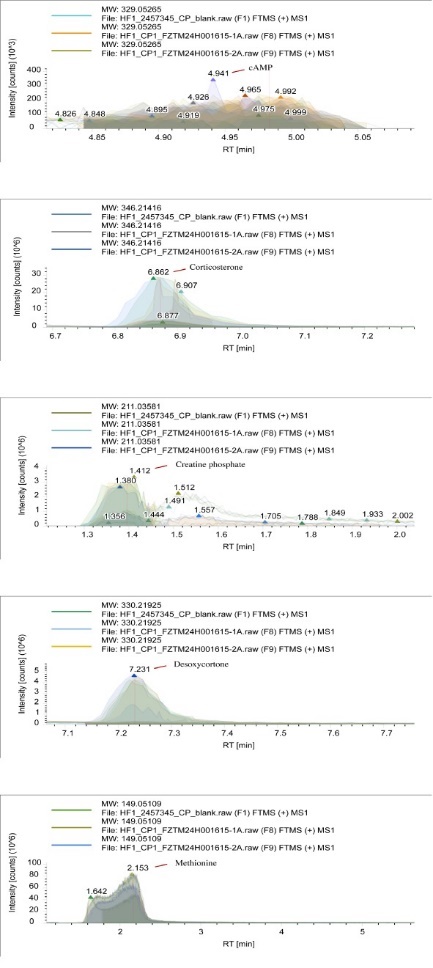


**Supplementary Figure 2:** Selected Metabolites, including cAMP, Corticosterone, Creatine phosphate, Desoxycortone, and Methionine, labeled on the Extracted ion chromatogram (XIC).

**1.2 Supplementary Tables**

**Supplementary Table 1:**

Supplementary Table 1: Content of Six Major Flavonoid Standards in SCFs

| **No** | **Name** | **Formula** | **tR(min)** | **CAS** | **Content** | **Wt%** |
| --- | --- | --- | --- | --- | --- | --- |
| 1 | Hyperoside | C_21_H_20_O_12_ | 10.63 | 482-36-0 | 84770.034 ug/g | 8.477 |
| 2 | Isoquercitrin | C_21_H_20_O_12_ | 11.33 | 482-35-9 | 13249.506 ug/g | 1.325 |
| 3 | Astragalin | C_21_H_20_O_11_ | 15.35 | 480-10-4 | 3024.968 ug/g | 0.302 |
| 4 | Quercetin | C_15_H_10_O_7_ | 20.13 | 117-39-5 | 274.642 ug/g | 0.027 |
| 5 | Kaempferol | C_15_H_10_O_6_ | 22.72 | 520-18-3 | 938.184 ug/g | 0.094 |
| 6 | Isorhamnetin | C_16_H_12_O_7_ | 23.25 | 480-19-3 | 96.465 ug/g | 0.010 |

**Supplementary Table 1:** Based on Supplementary Figure 1, the content and retention times of each compound were accurately measured using the LC-MS/MS system. Among the six major flavonoids in SCFs, hyperoside had the highest content at 84,770.034 µg/g (8.477%), followed by isoquercitrin at 13,249.506 µg/g (1.325%). Isorhamnetin had the lowest content at 96.465 µg/g (0.010%). The retention times (tR) ranged from 19.913 minutes (quercetin) to 33.68 minutes (isorhamnetin).

Supplementary Table 2: The SPF between the Ctrl and CUMS groups (‾*x±s* )

| group | sucrose preference rate % | | | | |
| --- | --- | --- | --- | --- | --- |
|  | Day0 | Day7 | Day14 | Day21 | Day28 |
| Ctrl | 85%±0.04 | 83%±0.05 | 82%±0.05 | 82%±0.09 | 82%±0.08 |
| CUMS | 83%±0.10 | 82%±0.10 | 79%±0.08 | 69%±0.18 | 50%±0.11^△△△^ |

All data were expressed as means ± SEM (n = 10). ^△^*p*<0.05，^△△^*p*<0.01，^△△△^*p*<0.001 vs. the Ctrl group

Supplementary Table 2 shows the sucrose preference rates before and during modeling. On days 7, 14, and 21, there were no significant differences between the two groups. However, after 28 days of modeling, the sucrose preference rate in the CUMS model group was significantly reduced compared to the Ctrl group (*p*<0.001), confirming the success of the CUMS modeling.

Supplementary Table 3: The OFT between the Ctrl and CUMS group (‾*x±s* )

| group | n | Total distance (cm) | Central residence time (s) |
| --- | --- | --- | --- |
| Ctrl | 10 | 2882.79 ±3840.75 | 8.59±4.05 |
| CUMS | 10 | 905.13±490.90^△△^ | 1.99±2.44^△△△^ |

^△^*p*<0.05，^△△^*p*<0.01，^△△△^*p*<0.001 vs. the Ctrl group

Supplementary Table 3 presents the results of the open field test (OFT): The total distance traveled by CUMS model mice was significantly reduced compared to the Ctrl group (*p*<0.01). Similarly, the central residence time was significantly decreased in CUMS mice (*p*<0.001) after 4-week of modeling.

Supplementary **Table** 4: The TST between the Ctrl and CUMS group (‾*x±s* )

| group | N | Immobility time（s） |
| --- | --- | --- |
| Ctrl | 10 | 167.50 ±33.57 |
| CUMS | 10 | 224.71±36.04^△△^ |

^△^*p*<0.05，^△△^*p*<0.01，^△△△^*p*<0.001 vs. the Ctrl group

Supplementary Table 4 shows the results of the tail suspension test: Compared to the Ctrl group, the immobility duration of the CUMS model group was significantly extended after 4-week of modeling (*p*<0.01), mimicking depressive-like behaviors of despair.

Supplementary **Table** 5: The FST between the Ctrl and CUMS group (‾*x±s* )

| group | N | Immobility time（s） |
| --- | --- | --- |
| Ctrl | 10 | 45.18±36.06 |
| CUMS | 10 | 97.63±46.30^△^ |

^△^*p*<0.05，^△△^*p*<0.01，^△△△^*p*<0.001 vs. the Ctrl group

Supplementary Table 5 shows the results of the forced swim test (FST). Compared to the Ctrl group, the immobility time of the CUMS model group was significantly prolonged in the forced swim test (*p*<0.05) after 4-week of modeling, mimicking the despair behavior characteristic of depressive-like behavior.

**Supplementary Table 6. Differential metabolites between the CUMS and Ctrl groups**

| **NO** | **Metabolite** | **t_R_(min)** | **Formula** | **Ionization mode** | **VIP  score** | **P  value** | **Fold  change** |
| --- | --- | --- | --- | --- | --- | --- | --- |
|  |  |  |  |  |  |  |  |
| 1 | PE O-18:0_22:4 | 11.655 | C_45_H_84_NO_7_P | NEG | 1.2556 | △△△ | 0.1048 |
| 2 | Uridine 5'-Diphospho-N-Acetylgalactosamine | 1.622 | C_17_H_27_N_3_O_17_P_2_ | NEG | 1.2817 | △△△ | 2.3926 |
| 3 | 2-{2-oxo-2-[(2-oxo-3-azepanyl)amino]ethoxy}acetic acid | 1.492 | C_10_H_16_N_2_O_5_ | POS | 1.1969 | △△△ | 2.2182 |
| 4 | PC 36:10_38:10 | 10.166 | C_82_H_124_NO_8_P | POS | 1.0185 | △△△ | 0.2906 |
| 5 | 6-Deoxy-D-glucose | 1.523 | C_6_H_12_O_5_ | NEG | 1.2304 | △△△ | 2.3323 |
| 6 | 4',7-Dihydroxyflavanone | 6.171 | C_15_H_12_O_4_ | POS | 1.0406 | △△△ | 4.3109 |
| 7 | D-Fructose 1,6-bisphosphate | 1.625 | C_6_H_14_O_12_P_2_ | POS | 1.0413 | △△△ | 0.4271 |
| 8 | Cer 16:0;2O/16:2;(3OH)(FA 20:4) | 11.944 | C_52_H_91_NO_5_ | NEG | 1.2376 | △△△ | 0.0999 |
| 9 | LPC O-17:0 | 10.93 | C_25_H_54_NO_6_P | POS | 1.1616 | △△△ | 0.4899 |
| 10 | N-Acetylaspartic acid | 2.04 | C_6_H_9_NO_5_ | NEG | 1.2593 | △△△ | 2.4131 |
| 11 | Chenodeoxycholic Acid | 7.864 | C_24_H_40_O_4_ | NEG | 1.2649 | △△△ | 2.8931 |
| 12 | 4-oxododecanedioic acid | 3.021 | C_12_H_20_O_5_ | POS | 1.2337 | △△△ | 0.0999 |
| 13 | Citicoline | 1.439 | C_14_H_26_N_4_O_11_P_2_ | POS | 1.2515 | △△△ | 3.4519 |
| 14 | Hexadecanamide | 10.445 | C_16_H_33_NO | POS | 1.3066 | △△△ | 0.4210 |
| 15 | 16(R)-HETE | 9.083 | C_20_H_32_O_3_ | POS | 1.2222 | △△△ | 2.2577 |
| 16 | 5-trans prostaglandin F2β | 377.23 | C_20_H_34_O_5_ | POS | 1.1786 | △△△ | 2.0949 |
| 17 | GDP | 1.644 | C_10_H_15_N_5_O_11_P_2_ | NEG | 1.2686 | △△△ | 3.5296 |
| 18 | LPE 20:5 | 9.059 | C_25_H_42_NO_7_P | NEG | 1.1612 | △△△ | 0.4909 |
| 19 | D-Ala-D-Ala | 1.511 | C_6_H_12_N_2_O_3_ | POS | 1.2844 | △△△ | 3.6116 |
| 20 | LPE 15:0 | 9.318 | C_20_H_42_NO_7_P | NEG | 1.1878 | △△△ | 4.3505 |
| 21 | Sphinganine | 8.643 | C_18_H_39_NO_2_ | POS | 1.2222 | △△△ | 2.8753 |
| 22 | N6-Succinyl Adenosine | 5.333 | C_14_H_17_N_5_O_8_ | POS | 1.2693 | △△△ | 2.2821 |
| 23 | S-Adenosylhomocysteine | 2.445 | C_14_H_20_N_6_O_5_S | POS | 1.3034 | △△△ | 3.0716 |
| 24 | Lactobionic acid | 4.941 | C_12_H_22_O_12_ | NEG | 1.2702 | △△△ | 2.0412 |
| 25 | 22(S)-Hydroxycholesterol | 9.667 | C_27_H_46_O_2_ | POS | 1.0092 | △△△ | 3.5134 |
| 26 | 3-[(4-chlorobenzyl)thio]-4-methyl-5-undecyl-4H-1,2,4-triazole | 2.461 | C_21_H_32_C_l_N_3_S | POS | 1.1178 | △△△ | 2.0426 |
| 27 | PC 18:0_18:1 | 8.824 | C_44_H_86_NO_8_P | POS | 1.2858 | △△△ | 0.0095 |
| 28 | Daidzein | 5.891 | C_15_H_10_O_4_ | POS | 1.1614 | △△△ | 3.0758 |
| 29 | 4-Methyl-5-thiazoleethanol | 5.27 | C_6_H_9_NOS | POS | 1.1873 | △△△ | 2.1134 |
| 30 | Methionine | 2.167 | C_5_H_11_NO_2_S | POS | 1.3105 | △△△ | 2.2501 |
| 31 | L-Palmitoylcarnitine | 10.217 | C_23_H_45_NO_4_ | POS | 1.1726 | △△△ | 0.1809 |
| 32 | FPH | 5.217 | C_20_H_25_N_5_O_4_ | POS | 1.2451 | △△△ | 2.6548 |
| 33 | Oleanolic acid | 8.481 | C_30_H_48_O_3_ | POS | 1.1712 | △△△ | 2.4210 |
| 34 | Octadecanamine | 9.314 | C_18_H_39_N | POS | 1.2489 | △△△ | 0.3417 |
| 35 | LLK | 8.841 | C_18_H_36_N_4_O_4_ | POS | 1.2487 | △△△ | 2.2350 |
| 36 | Paracetamol | 1.497 | C_8_H_9_NO_2_ | POS | 1.1108 | △△△ | 2.0302 |
| 37 | Lipoic acid | 1.931 | C_8_H_14_O_2_S_2_ | NEG | 1.3019 | △△△ | 2.8373 |
| 38 | Uridine 5'-diphosphogalactose | 1.617 | C_15_H_24_N_2_O_17_P_2_ | NEG | 1.2318 | △△△ | 4.1435 |
| 39 | Desoxycortone | 7.272 | C_21_H_30_O_3_ | POS | 1.2132 | △△△ | 0.2158 |
| 40 | glutathione disulfide | 2.924 | C_20_H_32_N_6_O_12_S_2_ | POS | 1.3118 | △△△ | 2.7131 |
| 41 | cAMP | 5.071 | C_10_H_12_N_5_O_6_P | POS | 1.2636 | △△△ | 3.1130 |
| 42 | Norbuprenorphine | 5.209 | C_25_H_35_NO_4_ | POS | 1.2826 | △△△ | 0.1464 |
| 43 | CAR 20:2 | 9.162 | C_27_H_50_NO_4_ | POS | 1.2490 | △△△ | 0.4072 |
| 44 | ST 24:2;O4 | 8.341 | C_24_H_38_O_4_ | NEG | 1.2276 | △△△ | 2.2855 |
| 45 | LPC 15:1-SN1 | 7.35 | C_23_H_46_NO_7_P | POS | 1.2188 | △△△ | 0.4802 |
| 46 | N-Acetyl-aspartic acid | 1.573 | C_6_H_9_NO_5_ | POS | 1.2899 | △△△ | 3.3934 |
| 47 | 2-(2-amino-3-methylbutanamido)-3-phenylpropanoic acid | 5.689 | C_14_H_20_N_2_O_3_ | POS | 1.2396 | △△△ | 2.0977 |
| 48 | Cytidine | 1.505 | C_9_H_13_N_3_O_5_ | POS | 1.1056 | △△△ | 2.5015 |
| 49 | Creatine phosphate | 1.402 | C_4_H_10_N_3_O_5_P | POS | 1.2996 | △△△ | 10.8267 |
| 50 | PC 18:0_18:0 | 9.666 | C_44_H_88_NO_8_P | POS | 1.2222 | △△△ | 0.0256 |
| 51 | S-Adenosylmethionine | 1.397 | C_15_H_22_N_6_O_5_S | POS | 1.2839 | △△△ | 5.5544 |
| 52 | Adenosine 5'-monophosphate | 1.813 | C_10_H_14_N_5_O_7_P | NEG | 1.1250 | △△△ | 0.4951 |
| 53 | L-Threonic acid-1,4-lactone | 2.193 | C_4_H_6_O_4_ | POS | 1.1836 | △△△ | 2.0374 |
| 54 | LPC 20:2 | 10.481 | C_28_H_54_NO_7_P | POS | 1.2510 | △△△ | 3.4869 |
| 55 | Propionyl-L-carnitine | 5.596 | C_10_H_19_NO_4_ | POS | 1.0306 | △△△ | 2.0968 |
| 56 | LPA 21:2 | 9.505 | C_24_H_45_O_7_P | NEG | 1.2718 | △△△ | 0.4148 |
| 57 | Asp-Phe | 5.253 | C_13_H_16_N_2_O_5_ | NEG | 1.2092 | △△△ | 2.1443 |
| 58 | δ-Ribono-1,4-lactone | 2.471 | C_5_H_8_O_5_ | NEG | 1.3100 | △△△ | 2.0595 |
| 59 | Adenine | 1.59 | C_5_H_5_N_5_ | POS | 1.3050 | △△△ | 2.9345 |
| 60 | C-8 Ceramide-1-phosphate | 10.518 | C_26_H_52_NO_6_P | POS | 1.3017 | △△△ | 0.3515 |
| 61 | 5-(benzyloxy)-2-(hydroxymethyl)-1,4-dihydropyridin-4-one | 6.073 | C_13_H_13_NO_3_ | POS | 1.0717 | △△△ | 2.2649 |
| 62 | 3-Nitro-L-Tyrosine | 1.491 | C_9_H_10_N_2_O_5_ | POS | 1.1262 | △△△ | 2.5320 |
| 63 | PE O-18:1_22:4 | 10.473 | C_45_H_82_NO_7_P | POS | 1.2720 | △△△ | 0.1598 |
| 64 | Ferulic acid | 5.93 | C_10_H_10_O_4_ | NEG | 1.1175 | △△△ | 2.5459 |
| 65 | Thr-Leu | 5.478 | C_10_H_20_N_2_O_4_ | POS | 1.2894 | △△△ | 2.3450 |
| 66 | Uridine | 2.655 | C_9_H_12_N_2_O_6_ | NEG | 1.2228 | △△△ | 2.3107 |
| 67 | Adenosine | 4.425 | C_10_H_13_N_5_O_4_ | POS | 1.3093 | △△△ | 2.4062 |
| 68 | Nor-9-carboxy-δ9-THC | 6.822 | C_21_H_28_O_4_ | POS | 1.2988 | △△△ | 0.1832 |
| 69 | Corticosterone | 6.914 | C_21_H_30_O_4_ | POS | 1.2538 | △△△ | 0.2861 |
| 70 | 4-[(2-cyclohex-1-enylethyl)amino]-2H-chromen-2-one | 5.165 | C_17_H_19_NO_2_ | POS | 1.2068 | △△△ | 2.1683 |
| 71 | 11-Deoxy prostaglandin F1β | 323.258 | C_20_H_36_O_4_ | POS | 1.2104 | △△△ | 2.6375 |
| 72 | Cytidine 5'-monophosphate (hydrate) | 1.482 | C_9_H_14_N_3_O_8_P | POS | 1.3027 | △△△ | 2.2134 |
| 73 | L-Glutathione (reduced) | 5.189 | C_10_H_17_N_3_O_6_S | NEG | 1.1528 | △△△ | 2.8306 |
| 74 | Stearamide | 10.993 | C_18_H_37_NO | POS | 1.1656 | △△△ | 0.4549 |
| 75 | Guanine | 5.056 | C_5_H_5_N_5_O | POS | 1.3011 | △△△ | 2.4273 |
| 76 | Genistein | 6.461 | C_15_H_10_O_5_ | NEG | 1.2681 | △△△ | 5.0854 |
| 77 | gamma-Glutamylmethionine | 5.245 | C_10_H_18_N_2_O_5_S | NEG | 1.2555 | △△△ | 2.0428 |
| 78 | Tetrahydrocorticosterone | 7.76 | C_21_H_34_O_4_ | NEG | 1.1046 | △△△ | 0.3938 |
| 79 | DRH | 5.306 | C_16_H_26_N_8_O_6_ | POS | 1.1451 | △△△ | 2.0309 |
| 80 | 4-Guanidinobutanoic acid | 1.943 | C_5_H_11_N_3_O_2_ | POS | 1.3084 | △△△ | 2.3581 |
| 81 | LPE 18:2 | 9.521 | C_23_H_44_NO_7_P | NEG | 1.2718 | △△△ | 0.4115 |
| 82 | DPK | 5.2 | C_15_H_26_N_4_O_6_ | POS | 1.2735 | △△△ | 3.0861 |
| 83 | LPC 20:1-SN1 | 11.035 | C_28_H_56_NO_7_P | POS | 1.0652 | △△△ | 0.4833 |
| 84 | Xanthosine | 4.175 | C_10_H_12_N_4_O_6_ | NEG | 1.3060 | △△△ | 0.4547 |
| 85 | Acetyl-L-carnitine | 2.035 | C_9_H_17_NO_4_ | POS | 1.3122 | △△△ | 2.3472 |
| 86 | Nootkatone | 8.502 | C_15_H_22_O | POS | 1.1463 | △△△ | 4.3027 |
| 87 | GPK | 5.233 | C_13_H_24_N_4_O_4_ | POS | 1.1926 | △△△ | 2.5580 |
| 88 | Monobenzyl phthalate | 6.161 | C_15_H_12_O_4_ | NEG | 1.2130 | △△△ | 3.9161 |
| 89 | Adenylosuccinic acid | 5.227 | C_14_H_18_N_5_O_11_P | POS | 1.2074 | △△△ | 2.0169 |
| 90 | Inosine | 5.049 | C_10_H_12_N_4_O_5_ | POS | 1.1800 | △△△ | 2.2715 |
| 91 | Stearic acid | 11.658 | C_18_H_36_O_2_ | NEG | 1.2506 | △△△ | 0.4917 |
| 92 | 1-[4-(1-adamantyl)phenoxy]-3-piperidinopropan-2-ol hydrochloride | 10.553 | C_24_H_35_NO_2_ | POS | 1.2976 | △△△ | 0.3954 |
| 93 | PE O-20:1_22:6 | 11.047 | C_47_H_82_NO_7_P | NEG | 1.1239 | △△△ | 0.2624 |
| 94 | Benzamidine | 1.33 | C_7_H_8_N_2_ | POS | 1.3118 | △△△ | 33.9109 |
| 95 | N-Tetradecanamide | 9.344 | C_14_H_29_NO | POS | 1.2612 | △△△ | 0.4136 |
| 96 | N-Oleoyl Glycine | 10.143 | C_20_H_37_NO_3_ | NEG | 1.2832 | △△△ | 0.4880 |
| 97 | L-Cysteine-glutathione gisulfide | 1.471 | C_13_H_22_N_4_O_8_S_2_ | POS | 1.3108 | △△△ | 2.3363 |
| 98 | S-Lactoyglutathione | 1.58 | C_13_H_21_N_3_O_8_S | NEG | 1.2592 | △△△ | 0.2297 |
| 99 | LPE O-18:3 | 9.864 | C_23_H_44_NO_6_P | NEG | 1.2836 | △△△ | 0.4129 |
| 100 | Lysops 22:5 | 10.284 | C_28_H_46_NO_9_P | NEG | 1.0786 | △△△ | 0.4682 |
| 101 | PC O-18:0 | 10.572 | C_26_H_54_NO_7_P | POS | 1.3043 | △△△ | 0.3399 |
| 102 | Phenylacetaldehyde | 5.974 | C_8_H_8_O | NEG | 1.1803 | △△△ | 2.2755 |
| 103 | HexCer 18:1;2O/22:1 | 8.851 | C_46_H_87_NO_8_ | POS | 1.2296 | △△△ | 0.1058 |
| 104 | Methandrostenolone | 9.634 | C_20_H_28_O_2_ | NEG | 1.2544 | △△△ | 0.4991 |
| 105 | T-2 Triol | 5.076 | C_20_H_30_O_7_ | POS | 1.0830 | △△△ | 2.5294 |
| 106 | LPC 14:0 | 9.143 | C_22_H_46_NO_7_P | NEG | 1.1620 | △△△ | 0.4716 |
| 107 | HLK | 5.168 | C_18_H_32_N_6_O_4_ | POS | 1.2647 | △△△ | 2.1720 |
| 108 | 4-morpholinobenzoic acid | 5.308 | C_11_H_13_NO_3_ | POS | 1.1591 | △△△ | 2.0005 |
| 109 | PC 19:0_19:1 | 10.872 | C_46_H_90_NO_8_P | POS | 1.1020 | △△△ | 0.0936 |
| 110 | 4-oxo-4-[(1-phenylethyl)amino]but-2-enoic acid | 5.138 | C_12_H_13_NO_3_ | POS | 1.2573 | △△△ | 2.2062 |
| 111 | beta-Estradiol 17-Acetate | 8.366 | C_20_H_26_O_3_ | NEG | 1.2555 | △△△ | 0.3710 |
| 112 | Tetrahydroaldosterone | 7.113 | C_21_H_32_O_5_ | POS | 1.2149 | △△△ | 2.0249 |

^△^*p*<0.05，^△△^*p*<0.01，^△△△^*p*<0.001 vs. the Ctrl group

**Supplementary Table 6:** OPLS-DA analysis resulted in 112 metabolites identified for the CUMS/Ctrl pair, based on a VIP > 1 in the S-Plot and a t-test *p*-value < 0.05 (with a fold change >2 or <0.50)

**Supplementary Table 7. Differential metabolites between the H-SCFs and CUMS groups**

| **NO** | **Metabolite** | **t_R_(min)** | **Formula** | **Ionization mode** | **VIP  score** | **P  value** | **Fold  change** |
| --- | --- | --- | --- | --- | --- | --- | --- |
|  |  |  |  |  |  |  |  |
| 1 | Dulcitol | 9.832 | CH_26_O_2_ | NEG | 1.3915 | *** | 3.0530 |
| 2 | 5-Phenylvaleric Acid | 7.571 | C_11_H_14_O_2_ | NEG | 1.3894 | *** | 0.2316 |
| 3 | Glutathione | 1.544 | C_10_H_17_N_3_O_6_S | NEG | 1.2394 | *** | 0.4877 |
| 4 | Methanandamide | 6.489 | C_23_H_39_NO_2_ | POS | 1.0389 | *** | 2.8195 |
| 5 | Chenodeoxycholic Acid | 7.864 | C_24_H_40_O_4_ | NEG | 1.2356 | *** | 0.4921 |
| 6 | 4-oxododecanedioic acid | 3.021 | C_12_H_20_O_5_ | POS | 1.3251 | *** | 0.0978 |
| 7 | N-Acetyl-L-carnosine | 7.592 | C_11_H_16_N_4_O_4_ | NEG | 1.3471 | *** | 2.9533 |
| 8 | (2E,4E)-N-(2-methylpropyl)deca-2,4-dienamide | 9.902 | C_14_H_25_NO | POS | 1.3235 | *** | 0.4461 |
| 9 | SDMA | 1.463 | C_8_H_18_N_4_O_2_ | POS | 1.3848 | *** | 2.0928 |
| 10 | Tetrahydrofuran fentanyl-d5 | 7.168 | C_24_H_30_N_2_O_2_ | POS | 1.2083 | *** | 0.4445 |
| 11 | Cryptotanshinone | 5.241 | C_19_H_20_O_3_ | POS | 1.1487 | *** | 2.6536 |
| 12 | D-Ala-D-Ala | 1.511 | C_6_H_12_N_2_O_3_ | POS | 1.2233 | *** | 2.4286 |
| 13 | 3-[(4-chlorobenzyl)thio]-4-methyl-5-undecyl-4H-1,2,4-triazole | 2.461 | C_21_H_32_C_l_N_3_S | POS | 1.3796 | *** | 2.3608 |
| 14 | N-benzoyl-N'-(2-chlorophenyl)thiourea | 2.497 | C_14_H_11_C_l_N_2O_S | POS | 1.0536 | *** | 2.0222 |
| 15 | Methionine | 2.167 | C_5_H_11_NO_2_S | POS | 1.4035 | *** | 2.7344 |
| 16 | D-δ-Tocopherol | 7.15 | C_27_H_46_O_2_ | POS | 1.1751 | *** | 2.6160 |
| 17 | L-Palmitoylcarnitine | 10.217 | C_23_H_45_NO_4_ | POS | 1.2390 | *** | 0.1841 |
| 18 | (2E,4E)-N-(2-methylpropyl)dodeca-2,4-dienamide | 8.104 | C_16_H_29_NO | POS | 1.2737 | *** | 0.2397 |
| 19 | Testosterone undecanoate | 8.489 | C_30_H_48_O_3_ | POS | 1.2808 | *** | 0.4698 |
| 20 | FPH | 5.217 | C_20_H_25_N_5_O_4_ | POS | 1.3566 | *** | 2.9465 |
| 21 | Octopine | 1.493 | C_9_H_18_N_4_O_4_ | POS | 1.3988 | *** | 2.3570 |
| 22 | Octadecanamine | 9.314 | C_18_H_39_N | POS | 1.3497 | *** | 0.1894 |
| 23 | Diphenylamine | 7.786 | C_12_H_11_N | POS | 1.2187 | *** | 2.3100 |
| 24 | Lipoic acid | 1.931 | C_8_H_14_O_2_S_2_ | NEG | 1.3895 | *** | 2.6993 |
| 25 | Desoxycortone | 7.272 | C_21_H_30_O_3_ | POS | 1.3106 | *** | 0.1023 |
| 26 | 1-Methylhistidine | 1.327 | C_7_H_11_N_3_O_2_ | POS | 1.3997 | *** | 0.4697 |
| 27 | cAMP | 5.071 | C_10_H_12_N_5_O_6_P | POS | 1.3857 | *** | 3.3252 |
| 28 | Norbuprenorphine | 5.209 | C_25_H_35_NO_4_ | POS | 1.3613 | *** | 0.1980 |
| 29 | CAR 20:2 | 9.162 | C_27_H_50_NO_4_ | POS | 1.3534 | *** | 0.2442 |
| 30 | Glu-Glu | 1.504 | C_10_H_16_N_2O_7 | NEG | 1.3864 | *** | 0.4359 |
| 31 | Angiotensin IV | 5.314 | C_40_H_54_N_8_O_8_ | POS | 1.3222 | *** | 2.2998 |
| 32 | Creatine phosphate | 1.402 | C_4_H_10_N_3_O_5_P | POS | 1.2160 | *** | 3.5504 |
| 33 | Phe-Phe | 5.892 | C_18_H_20_N_2_O_3_ | POS | 1.3610 | *** | 4.0730 |
| 34 | LPC 20:2 | 10.481 | C_28_H_54_NO_7_P | POS | 1.3076 | *** | 2.5600 |
| 35 | N-lactoyl-phenylalanine | 6.136 | C_12_H_15_NO_4_ | NEG | 1.3340 | *** | 2.0346 |
| 36 | Asp-Phe | 5.253 | C_13_H_16_N_2_O_5_ | NEG | 1.3093 | *** | 2.0181 |
| 37 | CAR 18:2 | 8.578 | C_25_H_46_NO_4_ | POS | 1.2181 | *** | 0.4552 |
| 38 | Argininosuccinic acid | 1.378 | C_10_H_18_N_4_O_6_ | POS | 1.3727 | *** | 2.1315 |
| 39 | Lysopa 16:0 | 7.172 | C_19_H_39_O_7_P | POS | 1.2504 | *** | 2.5460 |
| 40 | Dehydroepiandrosterone (DHEA) | 7.393 | C_19_H_28_O_2_ | POS | 1.1980 | *** | 2.2452 |
| 41 | Palmitic Acid | 7.371 | C_16_H_32_O_2_ | POS | 1.0942 | *** | 0.4479 |
| 42 | Nor-9-carboxy-δ9-THC | 6.822 | C_21_H_28_O_4_ | POS | 1.3871 | *** | 0.1591 |
| 43 | (2S)-4-Oxo-2-phenyl-3,4-dihydro-2H-chromen-7-yl beta-D-glucopyranoside | 7.721 | C_21_H_22_O_8_ | NEG | 1.3959 | *** | 2.1677 |
| 44 | Corticosterone | 6.914 | C_21_H_30_O_4_ | POS | 1.3581 | *** | 0.1253 |
| 45 | 3-Indoxyl sulphate | 5.554 | C_8_H_7_NO_4_S | NEG | 1.3822 | *** | 2.1028 |
| 46 | Adipic acid | 5.542 | C_6_H_10_O_4_ | NEG | 1.3725 | *** | 2.0743 |
| 47 | Stearamide | 10.993 | C_18_H_37_NO | POS | 1.2702 | *** | 0.4786 |
| 48 | Genistein | 6.461 | C_15_H_10_O_5_ | NEG | 1.2728 | *** | 7.8461 |
| 49 | 25-hydroxycholecalciferol | 7.048 | C_27_H_44_O_2_ | POS | 1.2008 | *** | 2.2747 |
| 50 | Tetrahydrocorticosterone | 7.76 | C_21_H_34_O_4_ | NEG | 1.3877 | *** | 0.1002 |
| 51 | PC 30:0 | 10.3 | C_38_H_76_NO_8_P | POS | 1.0427 | *** | 2.7079 |
| 52 | Valeric acid | 5.543 | C_5_H_10_O_2_ | NEG | 1.3972 | *** | 2.0378 |
| 53 | Eicosapentaenoic acid ethyl ester | 9.005 | C_22_H_34_O_2_ | POS | 1.3144 | *** | 0.1696 |
| 54 | CAR 22:4 | 9.048 | C_29_H_50_NO_4_ | POS | 1.3419 | *** | 0.3108 |
| 55 | Solvent blue 4 | 5.15 | C_33_H_33_N_3_O | POS | 1.3761 | *** | 2.1573 |
| 56 | Acetyl-L-carnitine | 2.035 | C_9_H_17_NO_4_ | POS | 1.4012 | *** | 2.1673 |
| 57 | GPK | 5.233 | C_13_H_24_N_4_O_4_ | POS | 1.2118 | *** | 2.9718 |
| 58 | Propionylcarnitine | 5.038 | C_10_H_19_NO_4_ | POS | 1.3732 | *** | 2.1006 |
| 59 | Deoxycholic acid | 8.71 | C_24_H_40_O_4_ | NEG | 1.2098 | *** | 0.4741 |
| 60 | All-Trans-13,14-Dihydroretinol | 7.123 | C_20_H_32_O | POS | 1.2099 | *** | 2.4422 |
| 61 | Sedanolide | 7.054 | C_12_H_18_O_2_ | POS | 1.2008 | *** | 2.2747 |
| 62 | Tetrahydrocortisone | 7.105 | C_21_H_32_O_5_ | POS | 1.1487 | *** | 0.1749 |
| 63 | Lysopc 16:2 (2N Isomer) | 8.856 | C_24_H_46_NO_7_P | POS | 1.2497 | *** | 0.4505 |
| 64 | S-Lactoyglutathione | 1.58 | C_13_H_21_N_3_O_8_S | NEG | 1.3456 | *** | 0.1884 |
| 65 | LPE O-18:3 | 9.864 | C_23_H_44_NO_6_P | NEG | 1.3726 | *** | 0.4251 |
| 66 | LPC 34:0 | 10.3 | C_42_H_86_NO_7_P | POS | 1.0246 | *** | 2.2138 |
| 67 | Adrenosterone | 6.594 | C_19_H_24_O_3_ | POS | 1.0596 | *** | 2.8251 |
| 68 | Methandrostenolone | 9.634 | C_20_H_28_O_2_ | NEG | 1.3288 | *** | 0.4559 |
| 69 | T-2 Triol | 5.076 | C_20_H_30_O_7_ | POS | 1.3757 | *** | 3.1496 |
| 70 | 3-hydroxy-1,5-diphenylpentan-1-one | 5.967 | C_17_H_18_O_2_ | POS | 1.2198 | *** | 2.0647 |
| 71 | Prostaglandin B1 | 7.973 | C_20_H_32_O_4_ | POS | 1.2466 | *** | 2.2127 |
| 72 | L-Kynurenine | 5.265 | C_10_H_12_N_2_O_3_ | POS | 1.2034 | *** | 0.4937 |

^*^*p*<0.05，^**^*p*<0.01，^***^*p*<0.001 vs. the CUMS group

**Supplementary Table 7:** OPLS-DA analysis resulted in 72 metabolites identified for the H-SCFs/CUMS pair, based on a VIP > 1 in the S-Plot and a t-test *p*-value < 0.05 (with a fold change >2 or <0.50)

**Supplementary Table 8.** KEGG Enrichment of cAMP Pathway Between Ctrl and CUMS groups

| **Pathway** | **Gene Ratio** | **Bg Ratio** | **p value** | **p.adjust** | **q value** | **Gene Name** | **Count** |
| --- | --- | --- | --- | --- | --- | --- | --- |
| cAMP signaling pathway | 3/34 | 6/193 | 0.0687 | 0.4111 | 0.3891 | cAMP/Adenosine 5'-monophosphate/Adenosine | 3 |

**Supplementary Table 8:** KEGG Enrichment Analysis of Differential Metabolites in the cAMP Pathway Between Ctrl and CUMS Groups
